# Unraveling the role of autophagy regulation in Crohn's disease: from genetic mechanisms to potential therapeutics

**DOI:** 10.1007/s44307-024-00021-z

**Published:** 2024-03-21

**Authors:** Ziyue Yuan, Jing Ye, Bo Liu, Lan Zhang

**Affiliations:** 1 Sichuan Engineering Research Center for Biomimetic Synthesis of Natural Drugs, School of Life Science and Engineering, Southwest Jiaotong University, Chengdu, 610031 China; 2 Department of Biotherapy, Cancer Center and State Key Laboratory of Biotherapy, West China Hospital, Sichuan University, Chengdu, 610041 China

**Keywords:** Autophagy, Autophagy-related genes, Crohn's disease, Small-molecule modulators

## Abstract

Autophagy serves as the primary intracellular degradation mechanism in which damaged organelles and self-cytoplasmic proteins are transported to the lysosome for degradation. Crohn's disease, an idiopathic chronic inflammatory disorder of the gastrointestinal tract, manifests in diverse regions of the digestive system. Recent research suggests that autophagy modulation may be a new avenue for treating Crohn's disease, and several promising small-molecule modulators of autophagy have been reported as therapeutic options. In this review, we discuss in detail how mutations in autophagy-related genes function in Crohn's disease and summarize the modulatory effects on autophagy of small-molecule drugs currently used for Crohn's disease treatment. Furthermore, we delve into the therapeutic potential of small-molecule autophagy inducers on Crohn's disease, emphasizing the prospects for development in this field. We aim to highlight the significance of autophagy modulation in Crohn's disease, with the aspiration of contributing to the development of more efficacious treatments that can alleviate their suffering, and improve their quality of life.

## Introduction

Crohn's disease, a chronic idiopathic inflammatory bowel disorder, is named after the American gastroenterologist Burrill B. Crohn. In 1932, he published a seminal paper outlining Crohn's disease symptoms for the first time: fever, diarrhea, and emaciation (Crohn et al. 1984). Crohn's disease can occur anywhere in the gastrointestinal tract, with recurrent transmural intestinal inflammation produced by immune responses, most commonly involving the terminal ileum in adults but the entire intestine in children (Alula and Theiss 2023). Although the etiology of Crohn's disease has not been defined, multiple studies have revealed that Crohn's disease may be linked to genetic susceptibility, environmental factors, immune system dysfunction, or a combination of these factors (Ruthruff 2007).

Autophagy is a vital cellular process that entails the recycling and degradation of superfluous or defective cellular components such as organelles and proteins (Mizushima and Komatsu 2011). The initiation of autophagy involves the formation of the phagophore, where the edges of isolation membranes elongate and encompass cytoplasmic cargos. Following the encapsulation of cytoplasmic cargos within the double-membrane structure, autophagosomes undergo maturation, resulting in the formation of a fully developed autophagosome structure. Subsequently, these autophagosomes fuse with late endosomes and lysosomes, initiating the degradation process of the enclosed cargos (Glick et al. 2010). Autophagy dysregulation has been linked to a variety of diseases, including cancer, infections, neurodegenerative disorders, and autoimmune diseases (Klionsky et al. 2021).

Single nucleotide polymorphisms (SNPs) in autophagy-associated genes have been identified as a susceptibility factor for Crohn's disease, providing the first indication that autophagy contributes to the genesis of the disease. This information was obtained by genome-wide association studies (GWAS) (Verstockt et al. 2018). One of the initial pieces of evidence came from the autophagy-related 16 like 1 (ATG16L1) study by Hampe et al., who discovered a statistically significant interaction for Crohn's disease risk between rs2241880, a coding SNP (T300A), and established nucleotide-binding oligomerization domain 2 (NOD2) susceptibility variants (*P* = 0.039) (Hampe et al. 2007). NOD2 was the first gene implicated in Crohn's disease susceptibility, and mutations in the NOD2 gene may lead to aberrant activation of the immune system, which can increase the likelihood of developing Crohn's disease (Hugot et al. 2001; Ogura et al. 2001). As research progresses, more and more autophagy-related Crohn's disease susceptibility genes have been identified, such as IRGM, ULK1, LRRK2, TLR4, etc. (Lapaquette et al. 2012a). In addition, the therapeutic effects of some conventional drugs used to treat Crohn's disease may be mediated in part by modulation of the autophagy pathway, further confirming the link between autophagy and Crohn's disease (Hooper et al. 2017). Furthermore, several autophagy modulators are proposed to possess potential therapeutic effects on Crohn's disease (Zhang and Liu 2019).

Thoroughly analyzing the impact of autophagy-related gene mutations on Crohn's disease will be beneficial in deepening our understanding of the intricate interactions between autophagy and this disease. Subsequently, we discussed in detail the effect of autophagy-related gene mutations in Crohn's disease, elucidating that autophagy modulation may be a novel approach to treating Crohn's disease. In this review, we also emphasize the autophagy modulation involved in conventional small-molecule drugs for treating Crohn's disease and introduce autophagy modulators with potential therapeutic effects on Crohn's disease, which will help us to develop more drugs to treat Crohn's disease.

## Overview of Crohn's disease

Crohn's disease is commonly recognized as an autoimmune disease marked by chronic inflammation in any area of the gastrointestinal tract (Beutler 2001). The disease's progression is devastating, and its prevalence is expanding globally (Roda et al. 2020). Crohn's disease’s clinical manifestation varies depending on the location of the disease, the intensity of inflammation, and the disease's behavior (Danese et al. 2015). Pain in the lower right abdomen, persistent diarrhea, and weight loss are common symptoms of the disease (Alula and Theiss 2023). Patients may also feel loss of appetite and malaise (Torres et al. 2017). For patients with colon involvement, rectal bleeding or bloody diarrhea may be the main manifestation. Furthermore, approximately one-third of individuals develop perianal disease (Eglinton et al. 2012). Lower health-related quality of life ratings are frequently associated with pain and fecal incontinence in those with perianal Crohn's disease (Parian et al. 2023). Extraintestinal manifestations, which can impact many body systems such as the eyes, skin, muscles, bones, mouth, and hepatobiliary system, are also reported in nearly half of Crohn's disease patients (Vavricka et al. 2011).

**Fig. 1:**
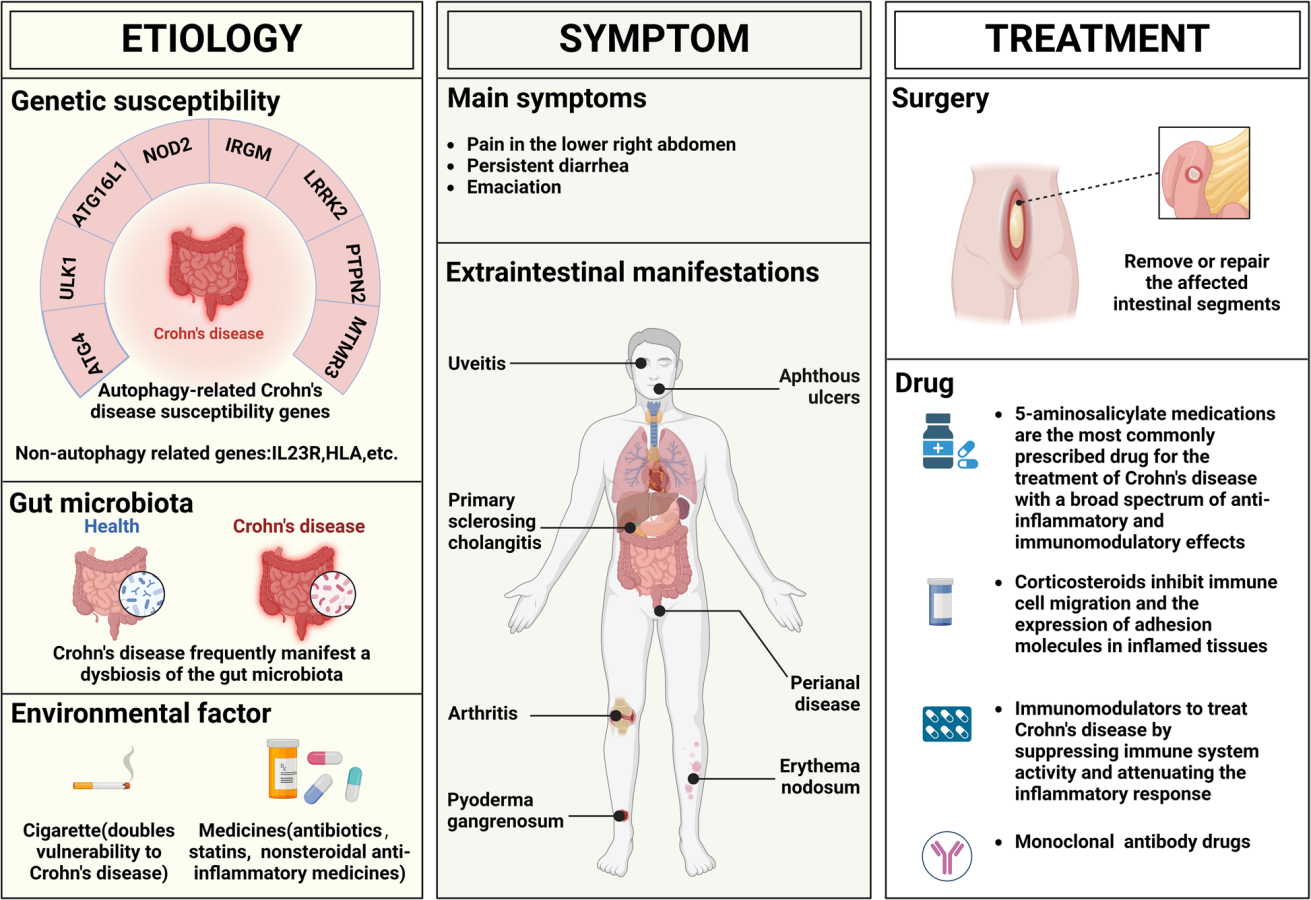
Overview of Crohn's disease. The etiology of Crohn's disease is related to genetic susceptibility, environmental factors, and gut microbiota. Genetic susceptibility is associated with autophagy-related genes, including ATG16L1, NOD2, IRGM, etc. Symptoms of Crohn's disease is determined by the site of onset, the intensity of inflammation, and the behavior of the disease, and can be classified into major symptoms and extraintestinal manifestations. Owing to the complexity of the underlying etiology, Crohn's disease is currently incurable, but it is possible to manage the patient's symptoms with medications and surgical procedures. The most used drugs in pharmacologic therapy fall into four categories: corticosteroids, immunomodulators, 5-ASA medications, and monoclonal antibody drugs

Although the exact etiology of Crohn's disease is unknown, it is believed that a combination of genetic susceptibility, environmental factors, and gut microbiota results in impaired epithelial barrier function and an abnormal immune response (Roda et al. 2020). GWAS has identified hundreds of loci associated with genetic susceptibility of Crohn's disease, including genes involved in innate immunity and bacterial sensing, such as ATG16L1, NOD2, IRGM, LRRK2, STAT3, IL23R, HLA, JAK2, and others (Sazonovs et al. 2022). Among them, ATG16L1, NOD2, IRGM, and LRRK2 are autophagy-associated genes, which highlights the key role of autophagy in genetic susceptibility of Crohn's disease (Alula and Theiss 2023). Environmental variables influence the onset and progression of Crohn's disease in genetically susceptible hosts. Smoking, one of the most studied environmental variables, doubles vulnerability to Crohn's disease (Mahid et al. 2006). The risk of Crohn's disease is further increased by early exposure to antibiotics (Ungaro et al. 2014). Statins (Ungaro et al. 2016) have been linked to a lower risk, while oral contraceptives (Cornish et al. 2008), aspirin (Ananthakrishnan et al. 2012), and nonsteroidal anti-inflammatory medicines are other medications that may raise risk. Micronutrients (zinc and iron) and vitamin D have also been linked to a higher risk of Crohn's disease (Ananthakrishnan 2015). Notably, individuals diagnosed with Crohn's disease frequently manifest a dysbiosis of the gut microbiota, and in particular, the adherent-invasive E coli (AIEC) pathotype is closely related to the pathogenesis of Crohn's disease (Palmela et al. 2018). This correlation underscores the significance of dysbiosis in the gut microbiota in the context of Crohn's disease development. Autophagy plays a role in modulating cellular responses to gut microbiota, and the anomalous proliferation of gut microbiota has the potential to initiate an immune response, thereby inducing inflammation and lesions. Paneth cells located at the base of the small intestinal crypts of Lieberkühn can clear invading pathogens and maintain a diverse gut microbiota by secreting secretory granules containing antimicrobial peptides (AMPs) and other peptides (Noah et al. 2011). Autophagy dysfunction of Paneth cell in Crohn's disease patients may lead to alterations in the composition of the gut microbiota, specifically manifested by the colonization of AIEC or Salmonella typhimurium on intestinal epithelial cells (IEC) (Garrett et al. 2010). Simultaneously, maintaining normal autophagic function is crucial for the production of pro-inflammatory cytokines in immune cells to sustain intestinal immune homeostasis. However, Crohn's disease patients with mutations in autophagy-related genes are unable to achieve this balance (Nguyen et al. 2013). These findings highlight that abnormal autophagy may be intertwined with multiple factors such as gut microbiota dysbiosis, immune system disorders, intestinal inflammation, and genetic mutations, which together contribute to the development of Crohn's disease. Because of the complexity of the underlying cause, Crohn's disease cannot currently be cured, but the patient's symptoms can be managed with the use of drugs, nutritional therapy, and surgical procedures. The most commonly used medications in pharmacotherapy are divided into four categories: corticosteroids (Dignass et al. 2010), immunomodulators (McDonald et al. 2014; Chande et al. 2015), 5-aminosalicylate (5-ASA) medications (Magro et al. 2020), and monoclonal antibody drugs (Chenna et al. 2023). Children with Crohn's disease can improve their symptoms through nutritional therapy because malnutrition and emaciation are often associated with this disease (Sasson et al. 2021). Surgery is indicated in a variety of scenarios, including structuring Crohn's disease with obstructive symptoms, fistulizing or perianal Crohn's disease with infectious complications or concerns linked to the draining of the fistula, and failure of medications (Feuerstein and Cheifetz 2017). All these medical interventions have their setbacks, even though they can all somewhat regulate Crohn's disease symptoms. For instance, immunomodulators can raise the risk of malignant tumors (Bourrier et al. 2016), corticosteroids can have adverse effects like diabetes, osteoporosis, high blood pressure, and infections (Uskudar Cansu et al. 2018), and the safety of upcoming monoclonal antibody drugs is unknown (Torres et al. 2017). Therefore, there is an imperative need to find innovative therapies to address the challenges of Crohn's disease. Our focus has recently centered on potential targets to modulate autophagy in treating Crohn's disease. MicroRNAs, particularly MicroRNA-106B and MicroRNA-143, have emerged as key players influencing intestinal autophagy and inflammatory responses by targeting susceptibility genes like ATG16L1 and ATG2B, potentially compromising autophagy-mediated bacterial clearance (Lu et al. 2014; Lin et al. 2018). Moreover, the 5-hydroxytryptamine (5-HT) receptor has emerged as a novel therapeutic target for Crohn's disease, as heightened intestinal 5-HT levels were found to inhibit autophagy and increase susceptibility to colitis (Haq et al. 2021).The discovery of these targets suggests that it is feasible to focus our research on the regulation of autophagy in Crohn's disease and reinforce our confidence in investigating the relationship between autophagy and Crohn's disease (Fig. 1).

## Regulation of autophagy

Autophagy is a multifaceted process that is separated into five stages: initiation, nucleation, extension, fusion, and degradation (Dikic and Elazar 2018). The activation of the Unc-51-like kinase 1 (ULK1) complex (consisting of ULK1, RB1-inducible coiled-coil protein 1 (FIP200), autophagy-related protein 13 (ATG13), and ATG101) is essential for autophagy initiation (Zachari and Ganley 2017). AMP-activated protein kinase (AMPK) directly activates ULK1 and induces autophagy under glucose starvation conditions by phosphorylating Ser317 and Ser777. In contrast, a rise in mTOR activity under nutritional conditions inhibits ULK1 activation because it leads to phosphorylation at Ser757 of ULK1, interrupting the connection between ULK1 and AMPK (Kim et al. 2011). To initiate phagophore nucleation, the ULK1 complex activates the class III PI3K (PI3KC3) complex I, which is made up of Beclin 1, ATG14, vacuolar protein sorting 34 (VPS34), activating molecule in Beclin 1-regulated autophagy protein 1 (AMBRA1), and general vesicular transport factor (p115) (Noda 2017; Zhou et al. 2017). When PI3KC complex I is activated, VPS34 generates phosphatidylinositol 3-phosphate (PI3P), facilitating the recruitment of autophagy-related PI3P-binding proteins such as WD-repeat protein that interact with PtdIns (WIPI) protein and Double FYVE-containing protein 1 (DFCP1) (Lamb et al. 2013). ATG9 vesicles were also recruited at this time to extend the phagophore (Shima et al. 2019). Furthermore, the extension of the phagophore membrane engages two ubiquitin-like systems, namely, the microtubule-associated protein light chain 3 (LC3) complex and the ATG12-ATG5-ATG16L1 complex (Itakura and Mizushima 2010). LC3-II plays a dual role in both elongating the phagophore membrane and sealing the phagophore (Jiang et al. 2021). Autophagosomes are double-layered vesicles that are produced when the phagophore membrane is sealed and eventually fuses with lysosomes. The autophagic cargo is digested by acidic hydrolases in the lysosome, and recycled nutrients are released back into the cytoplasm and utilized by the cell. Although autophagy was initially thought to be a non-selective process occurring under starvation conditions, it is now recognized that autophagy also plays a role in the targeted and selected removal of specific substrates, so-called selective autophagy, which includes processes such as mitophagy, xenophagy and aggrephagy. Selective autophagy encompasses distinct stages, involving the identification of a degradation signal, cargo recognition facilitated by selective autophagy receptors, ubiquitination, and the recruitment of autophagosomal machinery (Mancias and Kimmelman 2016).

Autophagy is a self-digestive process in which the cell clears unnecessary cellular components, such as damaged organelles or proteins, as their accumulation can be toxic to the entire system. Additionally, autophagy serves as a defense mechanism against invading pathogens, constituting an integral part of both innate and adaptive immunity (Azzman 2019). Its activation primarily occurs through sensors of innate immunity, namely pattern recognition receptor (PRR) signaling. The fundamental challenge faced by intestinal immunity is maintaining a delicate balance between tolerance and responsiveness to microbes. Research on polymorphisms in autophagy-related genes (ATG16L1, NOD2, IRGM) suggests that compromised sensing and handling of intracellular bacteria by innate immunity contribute to various inflammatory, immune, and metabolic disorders. Currently, autophagy is widely recognized as a key regulatory mechanism with the capacity to integrate multiple aspects of Crohn's disease pathogenesis (Muzes et al. 2013).

## Autophagy-related genes are associated with Crohn's disease susceptibility

A substantial body of evidence implies that more than 30 different genomic loci are linked to genetic susceptibility to Crohn's disease, including epidemiologic data based on family study concordance data, linkage analysis, and GWAS (Van Limbergen et al. 2009). The genes encoded by these loci are involved in many autophagy-related genes, such as ATG16L1, IRGM, LRRK2, etc. (Table 1). Therefore, elucidating the function that autophagy plays in Crohn's disease could consolidate our understanding of Crohn's disease and offer promising avenues for future research.

**Table 1:** Autophagy-related genes involved in susceptibility to Crohn's disease

Susceptibility gene	Role in autophagy	Crohn's disease-associated risk polymorphism	Functional impact of risk variant	Refs
ATG16L1	Involved in autophagosome membrane extension as part of the autophagy proteins ATG5 and ATG12 complexes	T300A (rs2241880)	Affected Paneth cell production of AMPs and macrophage and DCs over-secretion of PRR-mediated pro-inflammatory cytokines	(Kuma et al. 2002; Hampe et al. 2007; Rioux et al. 2007; Cadwell et al. 2008; Fujita et al. 2008; Saitoh et al. 2008; Takeuchi and Akira 2010; Strober and Fuss 2011; Ulm et al. 2012; Clevers and Bevins 2013; He et al. 2016; Hemshekhar et al. 2016; Ho et al. 2017; Samie et al. 2018; Lueschow and McElroy 2020; Honjo et al. 2021; Okai et al. 2022)
NOD2	Induction of autophagy by binding to ATG16L1	R702W (rs2066844) G908R (rs2066845) L1007fs (rs2066847)	Inhibition of autophagy and reduced MDP sensing leads to abnormal barrier function and bacterial clearance	(Hugot et al. 2001; Bonen et al. 2003; Abbott et al. 2007; Windheim et al. 2007; Hasegawa et al. 2008; Bertrand et al. 2009; Krieg et al. 2009; Cooney et al. 2010; Homer et al. 2010; Travassos et al. 2010; Damgaard et al. 2012; Fridh and Rittinger 2012; Grimes et al. 2012; Balasubramanian and Gao 2017; Mirkov et al. 2017; Mukherjee et al. 2019; Xu and Lei 2020; Ashton et al. 2023)
IRGM	Modulation of selective autophagy for antimicrobial defense	C313T (rs10065172) A 20-kb deletion upstream of the IRGM gene	Leading to differences in IRGM expression levels and abnormalities in the cellular initiation and maintenance of autophagy to combat recalcitrant intracellular bacteria	(Parkes et al. 2007; Taylor 2007; McCarroll et al. 2008; Bjorkoy et al. 2009; Lapaquette et al. 2010; Prescott et al. 2010; Brest et al. 2011; Lapaquette et al. 2012b; Chauhan et al. 2015; Lazarou et al. 2015; Medina et al. 2015; Akabane et al. 2016; Bravo-San Pedro et al. 2017; Kumar et al. 2018; Guo et al. 2020; Kumar et al. 2020; Iorio et al. 2021)
LRRK2	Stimulation of macrophages dependent on activation of Beclin 1	rs3761863	Unknown	(Franke et al. 2010; Liu et al. 2011, 2017; Manzoni et al. 2016; Yan and Liu 2017; Ridler 2018; Takagawa et al. 2018; Ahmadi Rastegar and Dzamko 2020)
ULK1	Crucial protein for the initiation of autophagy	rs12303764 rs3923716	Defective macrophage-mediated clearance of AIEC	(Ganley et al. 2009; Henckaerts et al. 2011; Randhawa et al. 2017; Alsaadi et al. 2019; Buisson et al. 2019)
ATG4	Processing of LC3 to promote autophagosome formation	rs5973822 rs7248026 rs2304165 rs10439163	Increased incidence of granulomas	(Brinar et al. 2012; Cabrera et al. 2013; Fernandez and Lopez-Otin 2015)
MTMR3	Making nascent autophagosomes smaller and reducing autophagic activity	rs713875	Increased NOD2-induced caspase-1 activation, NF-κB signaling, and cytokine secretion decreased PI3P thus inhibiting autophagy	(Franke et al. 2010; Taguchi-Atarashi et al. 2010; Vergne and Deretic 2010; Lahiri et al. 2015)
PTPN2	By inhibiting the activity of EGFR, mTOR activity is indirectly inhibited, thus promoting autophagy	rs2542151 rs1893217	Affected autophagosome formation and induced MDP-dependent MAPK phosphorylation	(Scharl et al. 2012a, 2012b; Song et al. 2022)

### ATG16L1

ATG16L1 forms an autophagy-important complex with ATG5 and ATG12 to localize the autophagosomes within the cell and to drive their elongation process (Kuma et al. 2002; Rioux et al. 2007; Fujita et al. 2008). ATG16L1 has a SNP that causes threonine to be replaced by alanine (T300A), which has been defined as a risk allele for Crohn's disease (Hampe et al. 2007; Rioux et al. 2007). The Crohn's disease-related ATG16L1 mutation T300A disrupts intestinal immune homeostasis by diminishing AMPs production in Paneth cells and over-secretion of the pattern recognition receptors (PRRs)-mediated pro-inflammatory cytokines by dendritic cells (DCs) and macrophages (Okai et al. 2022).

Schwalbe and Paneth provided the initial description of Paneth cells as columnar epithelial cells with noticeable eosinophilic granules in the late 1800s (Clevers and Bevins 2013). Paneth cells, found within the small intestinal crypts of Lieberkühn, are intricately specialized secretory epithelial cells. The dense granules made by Paneth cells are rich in AMPs and immunomodulatory proteins, which control the composition of the intestinal flora (Lueschow and McElroy 2020). AMPs induce bacterial cytoplasm to flow out of the cell by piercing the bacterial membrane and producing holes (Ulm et al. 2012; Hemshekhar et al. 2016; Ho et al. 2017). Cadwell et al. used lysozyme staining examinations to examine the effects of the ATG16L1 risk allele on Paneth cells. They found that the granule exocytosis pathway exhibited significant abnormalities in ATG16L1-deficient Paneth cells (Cadwell et al. 2008). Consequently, Paneth cell biology and distinct regulatory characteristics are modulated by ATG16L1, which in turn affects the intestinal epithelium of Crohn's disease patients.

**Fig. 2:**
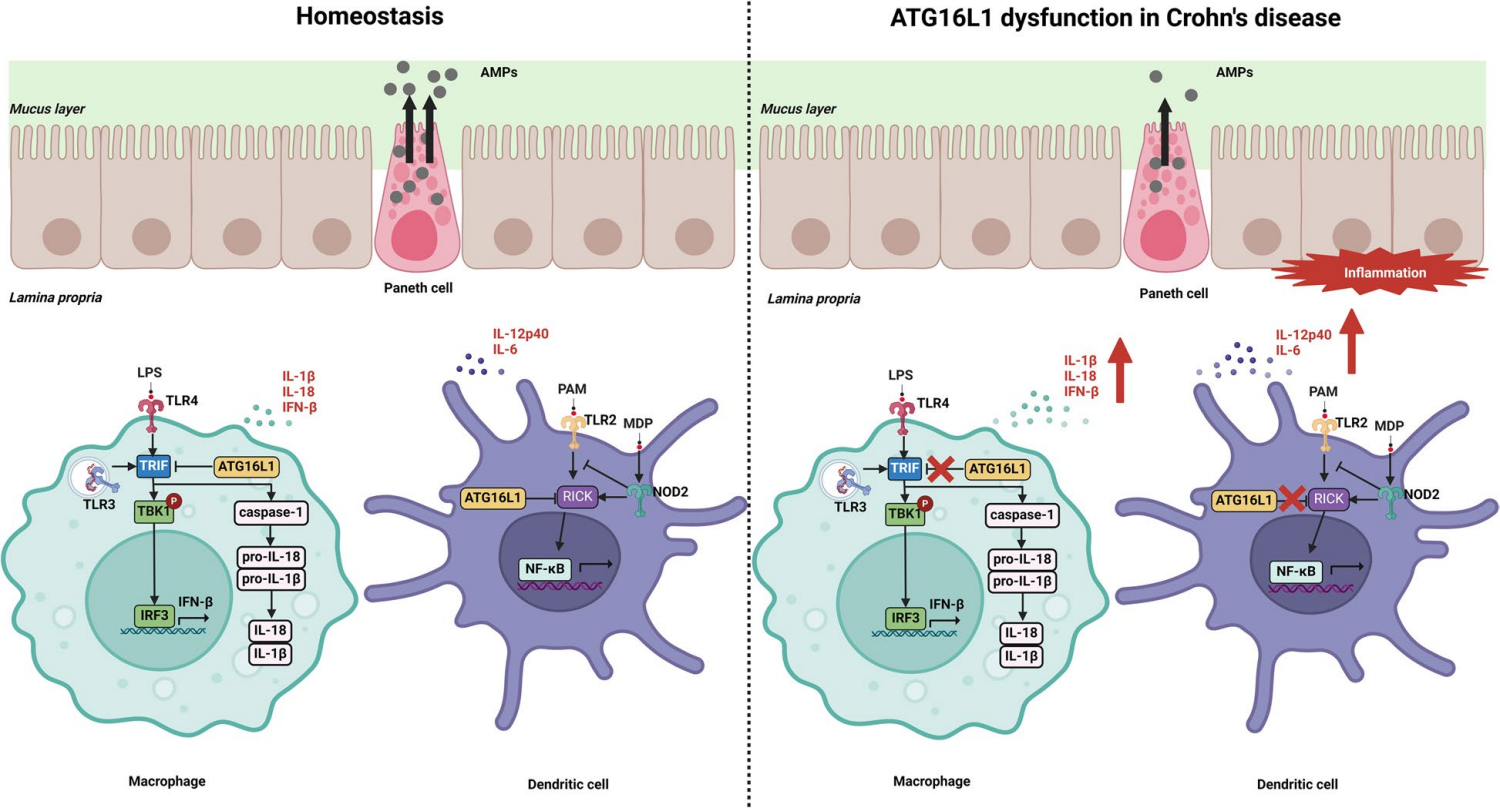
ATG16L1 dysfunction and increased risk of Crohn's inflammation. At homeostasis in vivo (left panel), the dense granules produced by Paneth cells are enriched with AMP and immunomodulatory proteins that control the composition of the intestinal flora. Moreover, after sensing the gut microbiota via PRRs, ATG16L1 negatively regulates TRIF- and RICK-mediated pro-inflammatory cytokine responses to maintain gut homeostasis. In the ATG16L1 dysfunctional state (right panel), AMP production by Paneth cells is reduced. This leads to increased PRRs stimulation of macrophages and DCs, which are not well regulated by ATG16L1, resulting in intestinal dysbiosis

Additionally, the progression of Crohn's disease is significantly influenced by pro-inflammatory cytokines (Strober and Fuss 2011). ATG16L1 is involved in PRRs-mediated signaling pathways in addition to autophagy (Fig. 2). PRRs are classified into four categories: Toll-like receptors (TLRs), C-type lectin receptors (CLRs), NOD-like receptors (NLRs), and RIG-I-like receptors (RLRs) (Takeuchi and Akira 2010). After recognition of gut microbiota by macrophages and DCs through PRRs, ATG16L1 negatively regulates their pro-inflammatory cytokine responses (Okai et al. 2022). ATG16L1-deficient macrophages, when stimulated with the TLR4 ligand lipopolysaccharide (LPS), release elevated levels of inflammatory cytokines IL-18 and IL-1β. The mechanism involves ATG16L1 deficiency inducing Toll/IL-1 receptor domain-containing adaptor inducing IFN-β (TRIF)-dependent caspase activation in LPS-stimulated macrophages (Saitoh et al. 2008). Caspase-1 subsequently cleaves IL-18 and IL-1β precursors, amplifying their production (He et al. 2016). A follow-up study revealed that macrophages isolated from patients carrying the Crohn's disease-associated ATG16L1 mutant T300A produced more IFN-β when stimulated with the ligand poly (I: C) for TLR3 and the ligand LPS for TLR4. Simultaneous activation of TLR3 and TLR4 leads to accumulation of TRIF, which subsequently leads to sustained kinase phosphorylation of tank-binding kinase 1 (TBK1) and transient phosphorylation of the TRIF-dependent transcription factor interferon regulatory factor 3 (IRF3), resulting in increased IFN-β production (Samie et al. 2018). It was also found that ATG16L1 negatively regulates the pro-inflammatory cytokine response mediated by receptor-interacting serine-threonine kinase (RICK), which is a subsequent signaling molecule for TLR2 and NOD2. TLR2 and RICK engage in a physical interaction in the presence of Pam3CSK4 (PAM), and ATG16L1 and RICK form a complex upon activation of NOD2 by muramyl dipeptide (MDP). MDP is considered to be the smallest immunogenic component of bacterial cell wall peptidoglycan (PGN) (Stafford et al. 2022). It has been demonstrated that, in response to PAM stimulation, cells transfected with ATG16L1 siRNA generated higher levels of nuclear factor-κB (NF-κB)-dependent cytokines, IL-6, and IL-12p40, than cells transfected with control siRNA (Honjo et al. 2021). Furthermore, MDP activation of NOD2 negatively controls TLR2-mediated pro-inflammatory factor responses in DCs (Watanabe et al. 2008). These results suggest that ATG16L1 plays an important role in the control of intestinal inflammation.

### NOD2

**Fig. 3:**
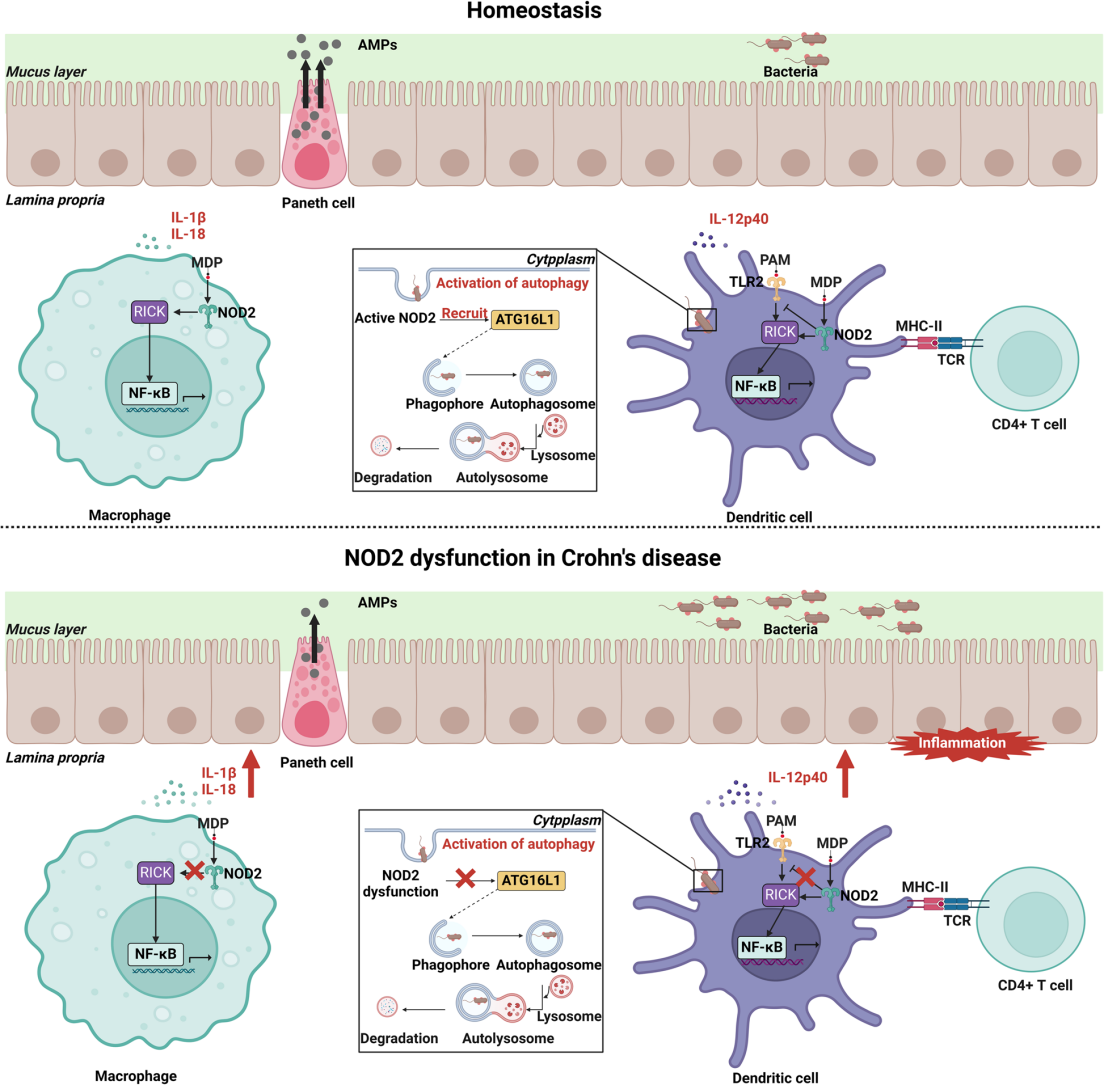
Regulation of the immune system and autophagic processes by NOD2. The activation of NOD2 promotes the sensing of MDP and autophagy in macrophages and DCs, mediating bacterial clearance, reducing inflammation, maintaining immune balance, and consequently preserving intestinal barrier function

Since the discovery of NOD2 variation in Crohn's disease in 2001, it has been recognized that NOD2 is the most important gene in the pathogenesis of Crohn's disease. The critical role of NOD2 in Crohn's disease has been confirmed by GWAS, next-generation sequencing, and functional analyses (Ashton et al. 2023). NOD2 is composed of two caspase recruitment domains (CARD), a nucleotide-binding domain (NBD), and a leucine-rich repeat (LRR) region that extends from the carboxyl to the amino terminus (Hugot et al. 2001). R702W (rs2066845), G908R (rs2066845), and L1007fs (rs2066847) were discovered to be three SNPs that are specifically linked to Crohn's disease and are located in or near the LRR region of NOD2 (Mirkov et al. 2017). NOD2 mutations associated with Crohn's disease interfere with several aspects of immune homeostasis, such as inhibition of autophagy and reduced MDP sensing in macrophages and DCs, as well as a reduction of AMPs secreted by Paneth cells, leading to abnormalities in barrier function and bacterial clearance (Fig. 3).

Activation of the NOD2 protein results in the formation of active oligomers that recruit adaptor proteins and generate downstream signaling cascades (Bonen et al. 2003). The LRRs region of NOD2 directly recognizes MDP (Grimes et al. 2012). MDP can enter cells via intracellular bacterial phagocytosis, solute channels, and endosomes. The NOD2 monomeric receptor responds to MDP by changing its conformation, which is controlled by ATP, and then oligomerizing through contacts with other exposed NBD domains. The downstream aptamer RICK is subsequently recruited and bound via the CARD-CARD interaction (Hasegawa et al. 2008; Fridh and Rittinger 2012). Then, X-linked IAP (XIAP), cellular inhibitors of apoptosis (cIAPs), and tumor necrosis factor receptor-associated factor 6 (TRAF6) are recruited to RICK and attached to it (Abbott et al. 2007; Bertrand et al. 2009; Krieg et al. 2009). The NF-κB essential modulator (NEMO) is ubiquitinated once the linear ubiquitin chain assembly complex (LUBAC) is recruited by the ubiquitin ligase XIAP (Damgaard et al. 2012). The subsequent phase involves recruitment and activation of the IκB kinase (IKK) complex, triggering phosphorylation of IκB (inhibitor protein of NF-κB). The expression of pro-inflammatory and AMP genes is then activated when NF-κB translocates to the nucleus (Balasubramanian and Gao 2017). In addition, transforming-growth-factorβ-activated kinase-1 (TAK1) is also recruited by polyubiquitinated RICK (Hasegawa et al. 2008). TAK1 functions by assembling with TAK1-binding proteins (TAB1, TAB2, and TAB3) (Xu and Lei 2020). TAK1 then activates mitogen-activated protein kinases (MAPKs), including extracellular signal-regulated kinase (ERK), p38α MAPK, and c-Jun N-terminal kinase (JNK). These kinases phosphorylate activator protein 1 (AP-1) transcription factors upon their translocation into the nucleus, which comprises ATF, c-fos, c-Jun, and JDP family members. The production of pro-inflammatory cytokines and AMPs is subsequently mediated by these transcription factors binding to TPA DNA-response elements (TRE) (Windheim et al. 2007; Mukherjee et al. 2019). NOD2 activation primes NLRP3 inflammasome activity by promoting NF-kB pro-inflammatory transcription, triggering downstream IL18 and IL1β production (Ashton et al. 2023). Furthermore, it has been shown that in NOD2-deficient mice, MDP stimulation leads to increased NF-κB activation (Maeda et al. 2005).

**Table 2:** Potential autophagy inducers for the treatment of Crohn's disease

Autophagy inducer	Chemical structure	Autophagy-related mechanism	Animal model	Refs
Celastrol		Blocking PI3K/Akt/mTOR signaling pathway	IL-10 deficient mice	(Zhao et al. 2015)
HU 308		Phosphorylation of AMPK-mTOR-P70S6K signaling cascade	DSS-induced colitis mice model	(Ke et al. 2016)
DHA		Inhibition of the mTOR pathway	IL-10 deficient mice	(Zhao et al. 2017)
Rapamycin		Inhibition of the mTOR pathway	IL-10 deficient mice HT-29 cells exposed to LPS TNBS chronic colitis mice	(Zhao et al. 2020a; Ni et al. 2022)
BRD5631		Repairing defects in bacterial co-localization with LC3	HeLa cells containing the Crohn's disease-associated allele ATG16L1 (T300A)	(Kuo et al. 2015)
Chlorpromazine		Restoration of autophagic flux defects	MDM	(Schwerd et al. 2017)
AMA0825	structure undisclosed	Significant decrease in p62 levels and an increase in the number of autophagosomes	DSS-induced colitis mice model	(Holvoet et al. 2017)
AC-73		Blocking activation of CD147/ERK1/2 and STAT3 signaling pathways	TNBS chronic colitis mice	(Butera et al. 2022)
Baicalin		Counteracting LPS-induced autophagy-related genes	HT-29 cells exposed to LPS	(Rizzo et al. 2021)

**Table 3:** Modulation of autophagy by current small-molecule drug for the treatment of Crohn's disease

Drug	Chemical structure	Modulation of Autophagy	Autophagy-related mechanism	Refs
Methylprednisolone		Inhibition of autophagy	Inhibition of autophagy via the PI3K/Akt/mTOR pathway	(Jang et al. 2022)
Budesonide		Inhibition of autophagy	Reduction of Beclin 1 and LC3 expression	(Maneechotesuwan et al. 2021)
Mesalazine		Induction of autophagy	Activation of the β1 subunit of macrophage AMPK	(Banskota et al. 2021)
Sulfasalazine		Induction of autophagy	Activation of the NF-κB/mTOR pathway	(Zhang et al. 2023)
Azathioprine		Induction of autophagy	Increasing the amount of LC3	(Morgan et al. 2019)
Methotrexate		Induction of autophagy	Enhancing the conversion of LC3-I to LC3-II	(Xiong et al. 2020)
Cyclosporine		Induction of autophagy	Protection of cells from ER stress and inhibits LC3-II expression	(Pallet et al. 2008)
Tacrolimus		Induction of autophagy	Upregulation of LC3 expression	(Xu et al. 2018; Yang et al. 2020)

Studies have revealed that NOD2 triggers autophagy activation in DCs, promoting major histocompatibility complex (MHC) class II antigen-specific CD4 + T cell responses and bacterial processing. Furthermore, it was discovered that the DCs derived from Crohn's disease patients who expressed NOD2 or ATG16L1 risk alleles associated with the disease were impaired in antigen presentation, bacterial transport, and autophagy induction (Cooney et al. 2010). In addition, another study showed that NOD2 can attract the autophagy protein ATG16L1 to the plasma membrane at the bacterial entry through a process that is not dependent on the transcription factor NF-κB or the adapter RICK. In pure cells with NOD2 shifter mutations associated with Crohn's disease, mutant NOD2 fails to recruit ATG16L1 to the plasma membrane, and autophagosome encapsulation of invading bacteria is impaired (Travassos et al. 2010). Moreover, it has been found that ATG16L1 and NOD2 both support the autophagy-dependent antimicrobial pathway, which is modified by Crohn's disease-associated mutations in a way that is specific to certain cell types (Homer et al. 2010). These findings demonstrate the connection between autophagy and two Crohn's disease-related susceptibility genes and support the involvement of NOD2 in impaired autophagic responses. Autophagy impairments can lead to an ineffective clearance of microbial infections, in turn exacerbates the inflammatory response, which may complicate the onset and progression of Crohn's disease and increase the severity of the disease.

### IRGM

**Fig. 4:**
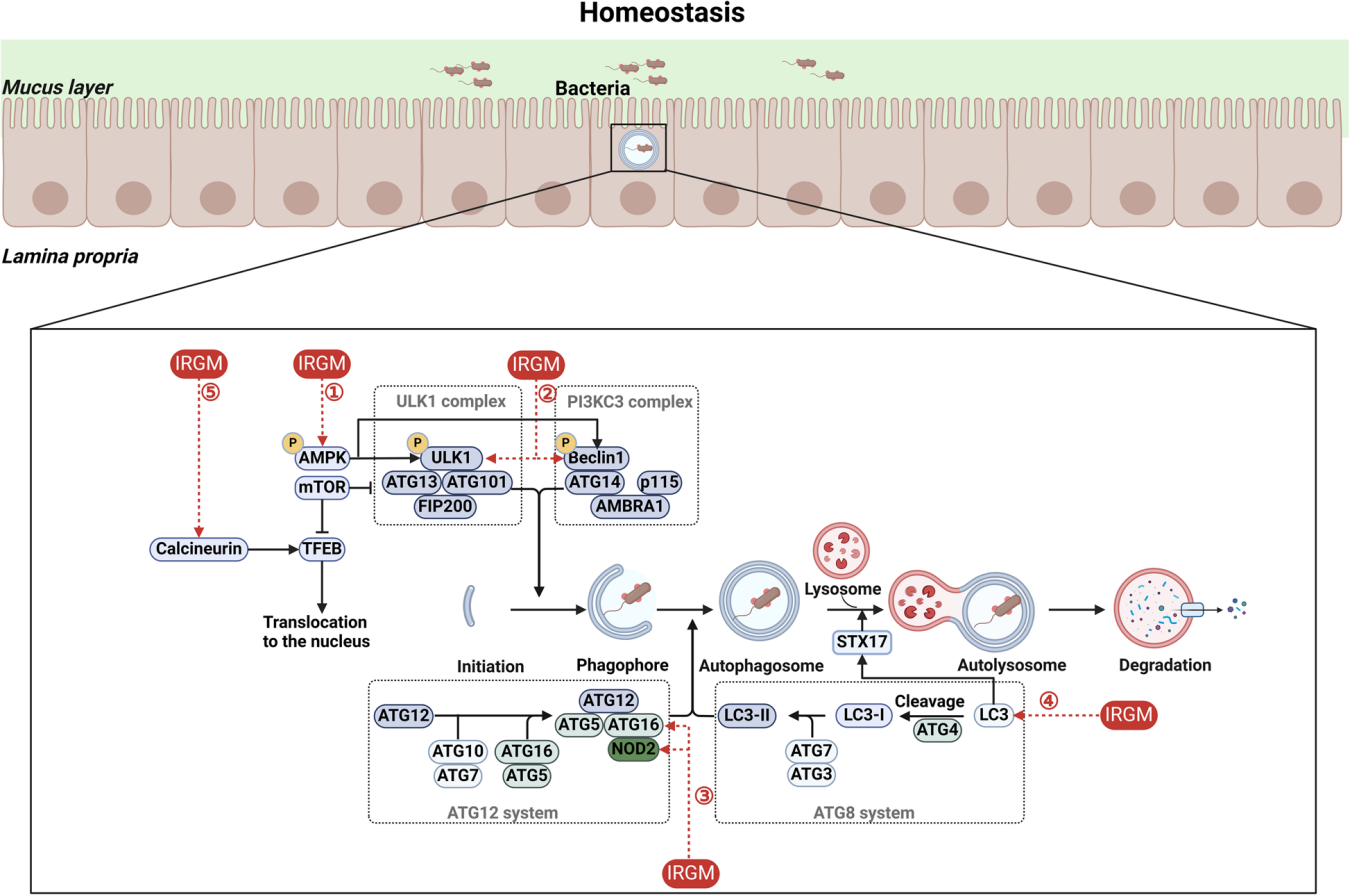
Regulation of core autophagy by IRGM. IRGM controls core autophagy and exerts its anti-microbial activities through five discrete but convergent mechanisms. The regulation of the core autophagic process by IRGM can be categorized into five aspects. (1) IRGM stimulates the phosphorylation of key autophagy regulators. (2) IRGM promotes the co-assembly of ULK1 and Beclin 1. (3) IRGM promotes the interaction between NOD2 and ATG16L1. (4) IRGM binds to LC3 and transports STX17 to autophagosomes for lysosomal fusion. (5) IRGM induces TFEB nuclear expansion by interacting with calcineurin

IRGM is a member of the immune-related GTPases (IRG) gene family and is located on chromosome 5q33.1 (Taylor 2007). IRGM can directly or indirectly regulate core autophagy (Fig. 4). The regulation of core autophagy by IRGM is multifaceted. IRGM controls core autophagy and exerts its anti-microbial activities through five discrete but convergent mechanisms. IRGM stimulates the phosphorylation of crucial autophagy regulators, assembling them into autophagy initiation complexes. It maintains AMPK in a phosphorylated state at Thr172 to promote its activation. Consistently, IRGM increases total activated ULK1 phosphorylated by AMPK at Ser317 and Ser555, as well as active Beclin 1 phosphorylated at Ser93 and Ser96 by AMPK and Ser15 by ULK1. Additionally, IRGM has the capacity to form complexes with ATG16L1 and NOD2, thereby enhancing the binding between ATG16L1 and NOD2 (Chauhan et al. 2015). NOD2 acts as a PRR that transduces microbial recognition signals to the IRGM, situating the IRGM in contact with various innate immune sensory inputs. Besides its role in autophagy initiation, IRGM can bind to LC3 and transport the SNAREcomponent STX17 to autophagosomes for lysosomal fusion (Kumar et al. 2018). IRGM has also been discovered to stimulate nuclear ectopic translocation of Transcription Factor EB (TFEB) via interacting with calcineurin, hence counteracting the action of mTOR as a negative regulator of TFEB (Kumar et al. 2020). TFEB is a protein that binds to the lysosomal periphery and is phosphorylated by mTOR. Under varying stress situations, it is maintained in the cytoplasm but can translocate to the nucleus and promote the expression of the lysosomal system (Medina et al. 2015). Once IRGM directed autophagy is set in motion, it clears microbes or their products, thus acting not only to contain infection but perhaps more importantly to prevent excessive inflammation (Chauhan et al. 2016).

Two IRGM polymorphisms, including a 'silent' SNP C313T (rs10065172) inside the coding region and a 20-kb deletion upstream of the IRGM gene, are strongly associated with Crohn's disease risk (Parkes et al. 2007; McCarroll et al. 2008; Prescott et al. 2010). Polymorphisms in the IRGM gene that are associated with Crohn's disease risk could alter expression levels and disrupt cellular functions crucial for initiating and sustaining autophagy against resilient intracellular bacteria. A study by Prescott et al. found that lower levels of IRGM expression were detected in untransformed lymphocytes from Crohn's disease patients (Prescott et al. 2010). Subsequently, heightened expression of the microRNA family miR-196 was detected in inflamed intestinal epithelial cells of individuals with Crohn's disease. This increased expression resulted in the reduction of protective IRGM variants, leaving the disease-associated variants unaffected. Consequently, the diminished levels of IRGM expression resulted in compromised autophagy and amplified intracellular bacterial replication (Brest et al. 2011). Studies have further demonstrated that induced knockdown of IRGM using siRNA in human cells leads to dysfunctional autophagy mechanisms, which in turn facilitate bacterial persistence and intensify pro-inflammatory responses (Lapaquette et al. 2010; Lapaquette et al. 2012b).

### LRRK2

**Fig. 5:**
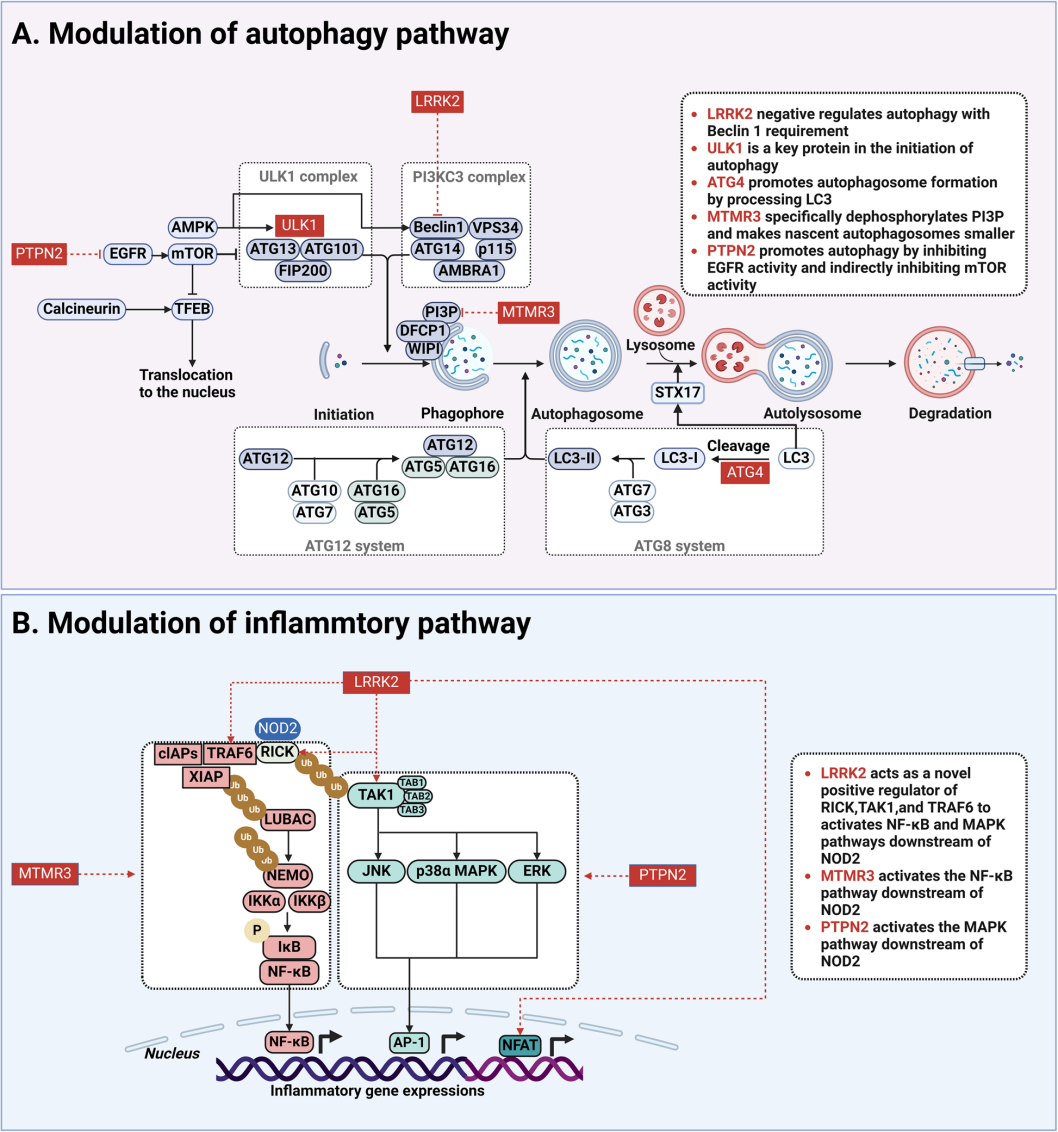
Other autophagy-related genes associated with Crohn's disease susceptibility. **A** Modulation of autophagy pathway. **B** Modulation of inflammatory pathway

Functional genetic variants in Leucine-rich repeat kinase 2 (LRRK2) are the largest known genetic contributor to Parkinson's disease, and these variants also increase the risk of Crohn's disease (Ridler 2018). A SNP (rs3761863) in LRRK2 has been found to be significantly associated with Crohn's disease susceptibility (Franke et al. 2010; Liu et al. 2011). In a noncanonical way that depends on Beclin 1 activation but is independent of mTOR and ULK1, chemical suppression of LRRK2 kinase activity promotes macroautophagy (Manzoni et al. 2016) (Fig. 5A). Apart from its involvement in autophagy, LRRK2 possesses the ability to influence innate immune responses and inflammatory pathways. LRRK2 is observed to be highly expressed in innate immune cells (Ahmadi Rastegar and Dzamko 2020). It can act as a novel positive regulator of RICK, TAK1, and TRAF6, activating downstream NF-κB and MAPK pathways through the NOD2-RICK pathway to promote the induction of inflammatory cytokines (Yan and Liu 2017; Takagawa et al. 2018). Another study showed that LRRK2 inhibits the activity of the transcription factor nuclear factor of activated T cells (NFAT), which activates the expression of inflammatory genes during transcription (Liu et al. 2011) (Fig. 5B). Furthermore, Paneth cell deficiencies are common in Crohn's disease patients, and LRRK2 is essential for maintaining Paneth cell function (Liu et al. 2017).

### ULK1

ULK1 is a serine/threonine protein kinase in mammals that functions by modulating the phosphorylation status of other autophagy-related proteins during the initiation process of autophagy (Ganley et al. 2009) (Fig. 5A). ULK1 phosphorylates ATG16L1 at Ser278, thereby facilitating autophagy in wild-type ATG16L1. This mechanism, on the other hand, increases ATG16L1 (T300A) degradation thus inhibiting autophagy (Alsaadi et al. 2019). Two SNPs in ULK1, rs12303764 and rs3923716 were significantly associated with Crohn's disease susceptibility (Henckaerts et al. 2011; Randhawa et al. 2017). Polymorphisms in ULK1 result in defective macrophage-mediated AIEC clearance in Crohn's disease patients (Buisson et al. 2019).

### ATG4

ATG4 is the only protease among dozens of ATG proteins, and since both the processing of LC3 by ATG4 and the deglutition reaction are important for the formation of autophagosomes, inhibition of ATG4 impedes autophagy at the stage of autophagosome formation (Fernandez and Lopez-Otin 2015) (Fig. 5A). It has been shown that ATG4B regulates intestinal homeostasis and protects mice from experimental colitis (Cabrera et al. 2013). Granulomas are a typical microscopic appearance in Crohn's disease and are linked to the autophagy gene variations ATG4A (rs5973822) and ATG4D (rs10439163; rs2304165; rs7248026) (Brinar et al. 2012).

### MTMR3

Myotubularin-related protein 3 (MTMR3) is a member of the myotubularin family and possesses an active phosphatase structural domain that specifically dephosphorylates PI3P (Vergne and Deretic 2010) (Fig. 5A). Local PI3P levels at autophagosome formation sites determine the initiation of autophagy and the size of autophagosome membrane structures. While overexpressing wild-type MTMR3 led to noticeably smaller nascent autophagosomes and decreased autophagic activity, MTMR3 knockdown boosted autophagosome formation (Taguchi-Atarashi et al. 2010). An SNP (rs713875) in MTMR3 was found to be significantly associated with Crohn's disease susceptibility by GWAS (Franke et al. 2010). One important study showed that the rs713875 Crohn's disease risk polymorphism enhanced MTMR3 expression, which in turn increased NOD2-induced caspase-1 activation, NF-κB signaling, and cytokine secretion, and lowered PI3P thus inhibiting autophagy (Lahiri et al. 2015) (Fig. 5B).

### PTPN2

Protein tyrosine phosphatase non-receptor 2 (PTPN2), also known as T cell protein tyrosine phosphatase (TCPTP), is a member of the PTP family of signaling proteins (Song et al. 2022). Tumor necrosis factor (TNF) and IFN-γ have been demonstrated to activate the Epidermal growth factor receptor (EGFR)-mTOR pathway, resulting in autophagosome formation and increased intracellular PTPN2 protein level. By suppressing EGFR activity and possibly indirectly suppressing mTOR activity, PTPN2 promotes autophagy (Fig. 5A). The presence of Crohn's disease-associated ATG16L1 (T300A) inhibits TNF and IFN-γ-induced PTPN2 protein increase. It has also been discovered that the Crohn's disease-associated PTPN2 variation (rs2542151) increases mTOR activity, which impairs autophagy (Scharl et al. 2012b). Another study showed that another SNP in PTPN2 (rs1893217) also influences autophagosome formation and induces NOD2-dependent MAPK phosphorylation (Scharl et al. 2012a) (Fig. 5B).

Overall, autophagy-related genes implicated in Crohn's disease have a significant role in the pathogenesis of the disease. Polymorphisms and mutations in these genes increase the genetic susceptibility to Crohn's disease, and further research is warranted to elucidate the specific roles of these genes in Crohn's disease and provide additional clues for treatment and prevention.

## Autophagy modulation for the treatment of Crohn's disease

Patients with Crohn's disease may require surgical or pharmacologic treatment, which treatment they receive depends on the severity of their disease or other clinical features, such as age of onset and major comorbidities (Cushing and Higgins 2021). Further studies of several drugs currently used to treat Crohn's disease have shown that they affect autophagy, such as anti-TNF drugs and thiopurines (Nys et al. 2013). In addition, there is a growing recognition of the potential therapeutic benefits of small-molecule autophagy modulators, including autophagy inducers and inhibitors, for the treatment of Crohn's disease. Currently, relevant studies on small-molecule autophagy inhibitors are very limited, and therefore, they are not discussed in this section.

### Therapeutic potential of small-molecule autophagy inducers in Crohn's disease

As mentioned previously, since autophagy-related genes are linked to Crohn's disease susceptibility, autophagy modulation could be an effective therapeutic intervention strategy. Therefore, it is necessary to summarize the current state of research on autophagy inducers in Crohn's disease (Table 2).

As a major regulator of cellular autophagy, mTOR is an attractive pharmacological target for the manipulation of autophagy, and it was originally identified as a target for rapamycin (Sabers et al. 1995). Rapamycin is a Food and Drug Administration (FDA) -approved drug and has shown some potential in the treatment of Crohn's disease in several small studies. For example, Massey et al. described the use of rapamycin in the treatment of perianal Crohn's disease in a 37-year-old woman, which resulted in significant symptom improvement (Massey et al. 2008). Celastrol, another autophagy inducer, was identified to induce autophagy in colonic tissues by blocking the PI3K/Akt/mTOR signaling pathway (Zhao et al. 2015). One week's administration of celastrol improves colitis in IL10-deficient mice, a murine model of Crohn's disease. IL-10, as an anti-inflammatory cytokine, plays an important role in maintaining intestinal immune homeostasis and preventing inflammatory responses, and the use of an IL10-deficient mouse model can mimic the disease of Crohn's disease. Subsequently, it was found that pre-treatment of peritoneal meacrophages with the selective cannabinoid receptor 2 (CB2R) agonist HU 308 promotes the phosphorylation of the AMPK-mTOR-P70S6K signaling cascade, which in turn enhances autophagic processes in peritoneal macrophages. This enhanced autophagy contributes to the inhibition of the initiation and activation of NLRP3 inflammatory vesicles and reduces the inflammatory cascade response, which ultimately mitigates DSS-induced colitis (Ke et al. 2016). In addition, docosahexaenoic acid (DHA) reduces the severity of chronic colitis in IL10-deficient mice by enhancing autophagy via mTOR pathway inhibition (Zhao et al. 2017).

Autophagy inducers that function on other pathways are as interesting as those that act on the mTOR signaling pathway. Kuo et al. identified a new small molecule autophagy regulator BRD5631 through high-throughput screening (Kuo et al. 2015). BRD5631 enhanced autophagy through an mTOR-independent pathway to clear invading bacteria and inhibit IL-1β production in HeLa cells containing the Crohn's disease-associated allele ATG16L1 (T300A). Chlorpromazine has also been used as an autophagy inducer and can restore autophagic flux defects. It was found that chlorpromazine treatment of human monocyte-derived macrophages (MDM) restored autophagic flux and overcame the bacterial killing deficiency in Niemann-Pick disease type C1 (NPC1). Early-onset IBD with granuloma formation and a Crohn's disease phenotype is predisposed by mutations in the NPC1 gene (Schwerd et al. 2017). In contrast to other autophagy inducers that have primarily been investigated in experimental studies, chlorpromazine has been approved by the FDA to treat psychosis (Plaze et al. 2021). However, there are still no clinical studies of chlorpromazine in treating Crohn's disease. Therefore, chlorpromazine is still classified as an autophagy inducer with therapeutic potential. Intestinal fibrosis-induced obstruction stands as a frequent complication in Crohn's disease, and Holvoet et al. evaluated the effect of locally acting Rho kinases (ROCK) inhibitors (AMA0825) on intestinal fibrosis. AMA0825-treated mice showed enhanced autophagy in intestinal fibroblasts, a significant decrease in p62 levels, and a rise in the number of autophagosomes, thereby preventing and reversing intestinal fibrosis (Holvoet et al. 2017). In addition to ROCK, another potential target for avoiding intestinal fibrosis is CD147, and the inhibitor of this protein, AC-73, may offer a promising new anti-fibrotic treatment option for Crohn's disease. Mice with chronic TNBS colitis exhibit higher levels of CD147 protein than control mice during the development of colon tissue fibrosis. By promoting autophagy and preventing the activation of the CD147/ERK1/2 and STAT3 signaling pathways, AC-73 treatment lowers intestinal fibrosis (Butera et al. 2022). Intestinal epithelial cells have an important barrier role in Crohn's disease. Rizzo et al. found that baicalin extracted from Scutellaria baicalensis root enhanced intestinal barrier function by inducing autophagy to counteract LPS-stimulation-induced down-regulation of LC3 and ATG5 gene expression in HT-29 cells (a human colonic epithelial cell line) (Rizzo et al. 2021). These findings highlight potential therapeutic implications for autophagy modulators in Crohn's disease, though further evidence is needed to establish their conventional treatment status.

### Modulation of autophagy by current small-molecule drug for the treatment of Crohn's disease

Current drug treatment options for Crohn's disease include traditional "step-up" therapy and newer "step-down" therapy. Traditional "step-up" therapy uses 5-ASA medications as first-line treatment, then corticosteroids as second-line treatment, and finally immunomodulators and anti-TNF antibodies as third-line treatment. "Step-down" therapy, on the other hand, allows for the use of immunomodulators and anti-TNF antibodies as initial treatment options for patients with severe Crohn's disease (Buchner et al. 2011). In this section, we will review the modulation of autophagy involved in small-molecule drugs currently used to treat Crohn's disease, as anti-TNF antibodies are not small-molecule drugs and will be excluded from this discussion (Table 3). Although a direct correlation between drug modulation of autophagy and disease improvement in patients with Crohn's disease has not been definitively established by current research, gaining a more in-depth understanding of the mechanisms of action of these drugs is of significant value. This is particularly crucial for the targeted and personalized application of these drugs within the Crohn's disease patient population.

5-ASA medications, such as sulfasalazine, mesalazine, olsalazine, and balsalazide, are among the most frequently prescribed treatments for Crohn's disease and exhibit a wide range of anti-inflammatory and immunomodulatory effects (Noureldin et al. 2021). A qualitative interview study of physicians with at least 4 years of independent experience treating patients with Crohn's disease showed that almost all physicians (93.3%) had prescribed 5-ASA medications for Crohn's disease patients and endorsed 5-ASA medications as the first-line treatment for Crohn's disease (Ma et al. 2018). One study in which mesalazine was able to act on the autophagy pathway caught our attention. Notably, mesalazine ameliorated dextran sulfate sodium (DSS)-induced colitis by activating the macrophage AMPK β1 subunit, enhancing autophagy, and causing a decrease in the production of macrophage proinflammatory factors (Banskota et al. 2021). In addition, sulfasalazine can induce autophagy via the NF-κB/mTOR pathway, thereby inhibiting neoplastic intimal hyperplasia after carotid artery injury in mice (Zhang et al. 2023).

Since many years ago, corticosteroids have been used to treat active Crohn's disease and continue to be the cornerstone treatment for recurring mild to severe Crohn's disease (Vavricka et al. 2014). Corticosteroids inhibit immune cell migration and the expression of adhesion molecules in inflamed tissues, as well as affecting the stability of messenger mRNA and down-regulating the transcription of genes involved in the production of pro-inflammatory cytokines (Yang and Lichtenstein 2002). Traditional corticosteroids like prednisone, prednisolone, and methylprednisolone are thought to be the most efficacious treatment for producing mild to moderate Crohn's disease remission (Malchow et al. 1984; Sandborn et al. 2007). Newer corticosteroids, such as budesonide, have the potential to minimize systemic medication and adverse effects while retaining efficacy (Vavricka et al. 2014). The FDA has approved delayed-release budesonide to treat mild to moderately active Crohn's disease (Chopra et al. 2006). Although corticosteroids have a limited effect on autophagy in Crohn's disease, studies on the relationship between corticosteroids and autophagy have never ceased. Recently, methylprednisolone and budesonide's modulatory effects on autophagy have been researched. Jang et al. showed that methylprednisolone could inhibit autophagy in rat femoral head vascular endothelial cells via the PI3K/Akt/mTOR pathway (Jang et al. 2022). By increasing autophagic flux, methylprednisolone, on the other hand, can encourage autophagy in osteoblasts (Zhao et al. 2020b). Additionally, in asthmatic patients, inhaling budesonide can inhibit the autophagy of airway macrophages by reducing the expression of LC3 and Beclin 1, and the inhibition of autophagy can increase the expression of IL-10, thereby controlling asthma inflammation (Maneechotesuwan et al. 2021).

The primary mechanism of action for immunomodulators in treating Crohn's disease involves the inhibition of immune system activity and the reduction of inflammation as well as the immune response, thereby effectively managing disease symptoms. Immunomodulators used to maintain Crohn's disease remission include thiopurines (6-mercaptopurine and azathioprine) and methotrexate (MTX) (Djuric et al. 2018). MTX is commonly considered an alternative therapeutic choice for individuals with Crohn's disease who have shown inadequate response to conventional thiopurine treatments (Savelkoul et al. 2022). Interestingly, these immunomodulators for maintaining remission in Crohn's disease also seem to affect the autophagic process. Morgan et al. investigated the effects of azathioprine on bone using a DSS model of colitis in mice and discovered that LC3 labeling was enhanced in the azathioprine-treated mice, implying autophagy induction (Morgan et al. 2019). In addition, by increasing the conversion of LC3-I to LC3-II, MTX was shown to activate autophagy in spermatocyte cells (Xiong et al. 2020). Other immunomodulators such as the calcineurin inhibitors cyclosporine and tacrolimus may help induce remission in severe Crohn's disease (Venner and Bernstein 2022). Tacrolimus and cyclosporine are both classified as calcineurin inhibitors, and tacrolimus has a more stable oral bioavailability and fewer side effects than cyclosporine for Crohn's disease (Chow and Leong 2007). Moreover, Hinterleitner et al. showed that the combination of cyclosporine, prednisolone, and azathioprine improved perianal fistulae in Crohn's disease (Hinterleitner et al. 1997). Our attention has also been drawn to the induction of autophagy by tacrolimus and cyclosporine. The induction of autophagy by cyclosporine was first described by Pallet et al. in 2008, who found that autophagosome production and LC3-II expression could be seen in primary cultured human renal tubular cells (Pallet et al. 2008). Tacrolimus induces autophagy by upregulating LC3 expression, which inhibits puromycin-induced podocyte injury in mice (Yang et al. 2020) and may protect vascular endothelial cells from low-density lipoprotein damage (Xu et al. 2018).

## Conclusion and perspective

Autophagy is essential for preserving intestinal homeostasis, regulating the relationship between immune responses and gut microbiota, and assisting the host in defending against intestinal pathogens (Mizushima 2018). Modulation of autophagy is an exciting therapeutic approach to treat Crohn's disease, and it has been shown that small-molecule drugs currently used for Crohn's disease can affect the autophagic pathway. However, it remains to be proven whether the autophagy regulation involved in these drugs is directly related to disease improvement. Given that these drugs have been clinically validated over a long period with proven safety and efficacy, it will become critical to investigate their direct impact on disease improvement in terms of autophagy regulation. In addition, a variety of small-molecule autophagy inducers have been recognized as potential therapeutic agents for Crohn's disease and have shown some therapeutic effects. However, the identified small-molecule autophagy inducers are still in the experimental stage, and further in-depth studies are still needed to determine whether they can enter into clinical applications, and the number of autophagy inducers with potential therapeutic effects is relatively limited, so it is imperative to search for more autophagy inducers. Notably, we discovered that starting from traditional Chinese medicine can give us fresh perspectives on how to discover small molecule autophagy inducers. A recent study found that the traditional Chinese herbal combination Xue-Jie-San (XJS) is beneficial in relieving clinical symptoms and aiding the repair of intestinal ulcers in Crohn's disease patients. Following therapy, XJS induces autophagy by blocking the mTOR/ULK1 signaling pathway. XJS also inhibits Notch1 and FGL1 signaling, which both affect mTOR signaling (Gao et al. 2023). In addition, Sanguisorba officinalis L. (SO) was able to suppress intestinal inflammation by promoting ATG7-dependent autophagy in colorectal macrophages (Yasueda et al. 2020). In the future, we can focus on extracting effective small-molecule components from traditional Chinese medicine, so as to better find small-molecule autophagy inducers with potential therapeutic effects. Although autophagy modulators show promise in the treatment of Crohn's disease, several challenges remain. First, we need a deeper understanding of the specific role and regulatory mechanisms of the autophagy pathway in the development of Crohn's disease. Second, since autophagy involves multiple signaling pathways and molecules, modulators targeting autophagy may have complex effects and side effects, and therefore more targeted and selective autophagy modulators need to be developed. In addition, the long-term safety and efficacy of autophagy modulators need to be further investigated.

In conclusion, this review provides an in-depth analysis of the impact of mutations in autophagy-related genes on Crohn's disease, offers a synthesis of the current understanding of how small-molecule drugs modulate autophagy in the treatment of this condition, and investigates the therapeutic promise of small-molecule autophagy inducers for Crohn's disease. We aim to highlight the significance of autophagy modulation in Crohn's disease, with the aspiration of contributing to the development of more efficacious treatments that can alleviate their suffering, and improve their quality of life.

## Data Availability

Not applicable.

## Abbreviations

AIEC: Adherent-invasive E. coli
AMBRA1: Activating molecule in Beclin 1-regulated autophagy protein 1
AMPK: AMP-activated protein kinase
AMP: Antimicrobial peptide
AP-1: Activator protein 1
ATG13: Autophagy-related protein 13
ATG16L1: Autophagy-related 16 like 1
CARD: Caspase recruitment domains
cIAPs: Cellular inhibitors of apoptosis
CLRs: C-type lectin receptors
DCs: Dendritic cells
DFCP1: Double FYVE-containing protein 1
DHA: Docosahexaenoic acid
DSS: Dextran sulfate sodium
EGFR: Epidermal growth factor receptor
ERK: Extracellular signal-regulated kinase
FDA: Food and Drug Administration
FIP200: RB1-inducible coiled-coil protein 1
GWAS: Genome-wide association studies
IKK: IκB kinase
IL: Interleukin
IRF3: Interferon regulatory factor 3
IRG: Immune-related GTPase
JNK: C-Jun N-terminal kinase
LC3: Light chain 3
LPS: Lipopolysaccharide
LRR: Leucine-rich repeat
LRRK2: Leucine-rich repeat kinase 2
LUBAC: Linear ubiquitin chain assembly complex
MAPK: Mitogen-activated protein kinase
MDM: Monocyte-derived macrophages
MDP: Muramyl dipeptide
MHC: Major histocompatibility complex
MMP: Mitochondrial membrane potential
MTMR3: Myotubularin-related protein 3
MTX: Methotrexate
NBD: Nucleotide-binding domain
NEMO: NF-κB essential modulator
NFAT: Nuclear factor of activated T cells
NF-κB: Nuclear factor-κB
NLRs: NOD-like receptors
NOD2: Nucleotide-binding oligomerization domain 2
NPC1: Niemann-Pick disease type C1
OMM: Outer mitochondrial membrane
p115: General vesicular transport factor
p62/SQSTM1: Sequestosome-1
PAM: Pam3CSK4
PARK2: Parkinson Disease Protein 2
PI3KC3: Class III PI3K
PI3P: Phosphatidylinositol 3-phosphate
PINK1: PTEN-induced putative kinase 1
PRRs: Pattern recognition receptors
PTPN2: Protein tyrosine phosphatase non-receptor 2
RICK: Receptor-interacting serine-threonine kinase
RLRs: RIG-I-like receptors
ROCK: Rho kinases
SNPs: Single nucleotide polymorphisms
SO: Sanguisorba officinalis L.
TAB: TAK1-binding proteins
TAK1: Transforming-growth-factorβ-activated kinase-1
TBK1: Tank-binding kinase 1
TCPTP: T cell protein tyrosine phosphatase
TFEB: Transcription Factor EB
TLRs: Toll-like receptor
TNBS: Trinitro-benzene-sulfonic acid
TNF: Tumor necrosis factor
TRAF6: Tumor necrosis factor receptor-associated factor6
TRE: TPA DNA-response elements
TRIF: Toll/IL-1 receptor domain-containing adaptor inducing IFN-β
ULK1: Unc-51-like kinase 1
VPS34: Vacuolar protein sorting 34
WIPI: WD-repeat protein that interact with PtdIns
XIAP: X-linked IAP
XJS: Xue-Jie-San
5-ASA: 5-Aminosalicylate
5-HT: 5-Hydroxytryptamine

## References

[CR1] Abbott DW, Yang Y, Hutti JE, Madhavarapu S, Kelliher MA, Cantley LC. Coordinated regulation of Toll-like receptor and NOD2 signaling by K63-linked polyubiquitin chains. Mol Cell Biol. 2007;27:6012–25. 17562858 10.1128/MCB.00270-07 PMC1952158

[CR2] Ahmadi Rastegar D, Dzamko N. Leucine rich repeat kinase 2 and innate immunity. Front Neurosci. 2020;14:193. 32210756 10.3389/fnins.2020.00193 PMC7077357

[CR3] Akabane S, Uno M, Tani N, Shimazaki S, Ebara N, Kato H, Kosako H, Oka T. PKA regulates PINK1 stability and parkin recruitment to damaged mitochondria through phosphorylation of MIC60. Mol Cell. 2016;62:371–84. 27153535 10.1016/j.molcel.2016.03.037

[CR4] Alsaadi RM, Losier TT, Tian W, Jackson A, Guo Z, Rubinsztein DC, Russell RC. ULK1-mediated phosphorylation of ATG16L1 promotes xenophagy, but destabilizes the ATG16L1 Crohn’s mutant. EMBO Rep. 2019;20:e46885. 31267703 10.15252/embr.201846885 PMC6607016

[CR5] Alula KM, Theiss AL. Autophagy in Crohn’s disease: converging on dysfunctional innate immunity. Cells. 2023;12(13):1779. 37443813 10.3390/cells12131779 PMC10341259

[CR6] Ananthakrishnan AN. Epidemiology and risk factors for IBD. Nat Rev Gastroenterol Hepatol. 2015;12:205–17. 25732745 10.1038/nrgastro.2015.34

[CR7] Ananthakrishnan AN, Higuchi LM, Huang ES, Khalili H, Richter JM, Fuchs CS, Chan AT. Aspirin, nonsteroidal anti-inflammatory drug use, and risk for Crohn disease and ulcerative colitis: a cohort study. Ann Intern Med. 2012;156:350–9. 22393130 10.1059/0003-4819-156-5-201203060-00007 PMC3369539

[CR8] Ashton JJ, Seaby EG, Beattie RM, Ennis S. NOD2 in Crohn’s disease-Unfinished Business. J Crohns Colitis. 2023;17:450–8. 36006803 10.1093/ecco-jcc/jjac124 PMC10069614

[CR9] Azzman N. Crohn’s disease: potential drugs for modulation of autophagy. Medicina (kaunas). 2019;55(6):224. 31146413 10.3390/medicina55060224 PMC6630681

[CR10] Balasubramanian I, Gao N. From sensing to shaping microbiota: insights into the role of NOD2 in intestinal homeostasis and progression of Crohn’s disease. Am J Physiol Gastrointest Liver Physiol. 2017;313:G7–13. 28450278 10.1152/ajpgi.00330.2016 PMC5538831

[CR11] Banskota S, Wang H, Kwon YH, Gautam J, Gurung P, Haq S, Hassan FMN, Bowdish DM, Kim JA, Carling D, Fullerton MD, Steinberg GR, Khan WI. Salicylates ameliorate intestinal inflammation by activating macrophage AMPK. Inflamm Bowel Dis. 2021;27:914–26. 33252129 10.1093/ibd/izaa305 PMC8128406

[CR12] Bertrand MJ, Doiron K, Labbe K, Korneluk RG, Barker PA, Saleh M. Cellular inhibitors of apoptosis cIAP1 and cIAP2 are required for innate immunity signaling by the pattern recognition receptors NOD1 and NOD2. Immunity. 2009;30:789–801. 19464198 10.1016/j.immuni.2009.04.011

[CR13] Beutler B. Autoimmunity and apoptosis: the Crohn’s connection. Immunity. 2001;15:5–14. 11485733 10.1016/s1074-7613(01)00176-5

[CR14] Bjorkoy G, Lamark T, Pankiv S, Overvatn A, Brech A, Johansen T. Monitoring autophagic degradation of p62/SQSTM1. Methods Enzymol. 2009;452:181–97. 19200883 10.1016/S0076-6879(08)03612-4

[CR15] Bonen DK, Ogura Y, Nicolae DL, Inohara N, Saab L, Tanabe T, Chen FF, Foster SJ, Duerr RH, Brant SR, Cho JH, Nunez G. Crohn’s disease-associated NOD2 variants share a signaling defect in response to lipopolysaccharide and peptidoglycan. Gastroenterology. 2003;124:140–6. 12512038 10.1053/gast.2003.50019

[CR16] Bourrier A, Carrat F, Colombel JF, Bouvier AM, Abitbol V, Marteau P, Cosnes J, Simon T, Peyrin-Biroulet L, Beaugerie L, group, C.s. Excess risk of urinary tract cancers in patients receiving thiopurines for inflammatory bowel disease: a prospective observational cohort study. Aliment Pharmacol Ther. 2016;43:252–61. 26549003 10.1111/apt.13466

[CR17] Bravo-San Pedro JM, Kroemer G, Galluzzi L. Autophagy and mitophagy in cardiovascular disease. Circ Res. 2017;120:1812–24. 28546358 10.1161/CIRCRESAHA.117.311082

[CR18] Brest P, Lapaquette P, Souidi M, Lebrigand K, Cesaro A, Vouret-Craviari V, Mari B, Barbry P, Mosnier JF, Hebuterne X, Harel-Bellan A, Mograbi B, Darfeuille-Michaud A, Hofman P. A synonymous variant in IRGM alters a binding site for miR-196 and causes deregulation of IRGM-dependent xenophagy in Crohn’s disease. Nat Genet. 2011;43:242–5. 21278745 10.1038/ng.762

[CR19] Brinar M, Vermeire S, Cleynen I, Lemmens B, Sagaert X, Henckaerts L, Van Assche G, Geboes K, Rutgeerts P, De Hertogh G. Genetic variants in autophagy-related genes and granuloma formation in a cohort of surgically treated Crohn’s disease patients. J Crohns Colitis. 2012;6:43–50. 22261526 10.1016/j.crohns.2011.06.008

[CR20] Buchner AM, Blonski W, Lichtenstein GR. Update on the management of Crohn’s disease. Curr Gastroenterol Rep. 2011;13:465–74. 21792543 10.1007/s11894-011-0220-x

[CR21] Buisson A, Douadi C, Ouchchane L, Goutte M, Hugot JP, Dubois A, Minet-Quinard R, Bouvier D, Bommelaer G, Vazeille E, Barnich N. Macrophages inability to mediate adherent-invasive E. coli replication is linked to autophagy in Crohn’s disease patients. Cells. 2019;8(11):1394. 31694333 10.3390/cells8111394 PMC6912674

[CR22] Butera A, Quaranta MT, Crippa L, Spinello I, Saulle E, Di Carlo N, Campanile D, Boirivant M, Labbaye C. CD147 targeting by AC-73 induces autophagy and reduces intestinal fibrosis associated with TNBS chronic colitis. J Crohns Colitis. 2022;16:1751–61. 35833587 10.1093/ecco-jcc/jjac084 PMC9683082

[CR23] Cabrera S, Fernandez AF, Marino G, Aguirre A, Suarez MF, Espanol Y, Vega JA, Laura R, Fueyo A, Fernandez-Garcia MS, Freije JM, Kroemer G, Lopez-Otin C. ATG4B/autophagin-1 regulates intestinal homeostasis and protects mice from experimental colitis. Autophagy. 2013;9:1188–200. 23782979 10.4161/auto.24797 PMC3748191

[CR24] Cadwell K, Liu JY, Brown SL, Miyoshi H, Loh J, Lennerz JK, Kishi C, Kc W, Carrero JA, Hunt S, Stone CD, Brunt EM, Xavier RJ, Sleckman BP, Li E, Mizushima N, Stappenbeck TS, Virgin HWt. A key role for autophagy and the autophagy gene Atg16l1 in mouse and human intestinal Paneth cells. Nature. 2008;456:259–63. 18849966 10.1038/nature07416 PMC2695978

[CR25] Chande N, Patton PH, Tsoulis DJ, Thomas BS, MacDonald JK. Azathioprine or 6-mercaptopurine for maintenance of remission in Crohn’s disease. Cochrane Database Syst Rev. 2015;2015:CD000067. 26517527 10.1002/14651858.CD000067.pub3 PMC9578512

[CR26] Chauhan S, Mandell MA, Deretic V. IRGM governs the core autophagy machinery to conduct antimicrobial defense. Mol Cell. 2015;58:507–21. 25891078 10.1016/j.molcel.2015.03.020 PMC4427528

[CR27] Chauhan S, Mandell MA, Deretic V. Mechanism of action of the tuberculosis and Crohn disease risk factor IRGM in autophagy. Autophagy. 2016;12:429–31. 26313894 10.1080/15548627.2015.1084457 PMC4835981

[CR28] Chenna VSH, Nagi TK, Suarez ZK, Hernandez OL, Nageye ME, Reyaz N, Reyaz I, Ali N. Comparison of effectiveness and safety of ustekinumab and adalimumab as induction or maintenance therapy in patients with moderate to severe Crohn’s disease: a systematic review and meta-analysis. Cureus. 2023;15:e38277. 37255887 10.7759/cureus.38277 PMC10226155

[CR29] Chopra A, Pardi DS, Loftus EV Jr, Tremaine WJ, Egan LJ, Faubion WA, Hanson KA, Johnson TA, Sandborn WJ. Budesonide in the treatment of inflammatory bowel disease: the first year of experience in clinical practice. Inflamm Bowel Dis. 2006;12:29–32. 16374255 10.1097/01.mib.0000192323.82426.83

[CR30] Chow DK, Leong RW. The use of tacrolimus in the treatment of inflammatory bowel disease. Expert Opin Drug Saf. 2007;6:479–85. 17877436 10.1517/14740338.6.5.479

[CR31] Clevers HC, Bevins CL. Paneth cells: maestros of the small intestinal crypts. Annu Rev Physiol. 2013;75:289–311. 23398152 10.1146/annurev-physiol-030212-183744

[CR32] Cooney R, Baker J, Brain O, Danis B, Pichulik T, Allan P, Ferguson DJ, Campbell BJ, Jewell D, Simmons A. NOD2 stimulation induces autophagy in dendritic cells influencing bacterial handling and antigen presentation. Nat Med. 2010;16:90–7. 19966812 10.1038/nm.2069

[CR33] Cornish JA, Tan E, Simillis C, Clark SK, Teare J, Tekkis PP. The risk of oral contraceptives in the etiology of inflammatory bowel disease: a meta-analysis. Am J Gastroenterol. 2008;103:2394–400. 18684177 10.1111/j.1572-0241.2008.02064.x

[CR34] Crohn BB, Ginzburg L, Oppenheimer GD, Landmark article Oct 15, 1932. Regional ileitis. A pathological and clinical entity. By Burril B. Crohn, Leon Ginzburg, and Gordon D. Oppenheimer. JAMA. 1984;251:73-9 10.1001/jama.251.1.73 6361290

[CR35] Cushing K, Higgins PDR. Management of Crohn disease: a review. JAMA. 2021;325:69–80. 33399844 10.1001/jama.2020.18936 PMC9183209

[CR36] Damgaard RB, Nachbur U, Yabal M, Wong WW, Fiil BK, Kastirr M, Rieser E, Rickard JA, Bankovacki A, Peschel C, Ruland J, Bekker-Jensen S, Mailand N, Kaufmann T, Strasser A, Walczak H, Silke J, Jost PJ, Gyrd-Hansen M. The ubiquitin ligase XIAP recruits LUBAC for NOD2 signaling in inflammation and innate immunity. Mol Cell. 2012;46:746–58. 22607974 10.1016/j.molcel.2012.04.014

[CR37] Danese S, Fiorino G, Mary JY, Lakatos PL, D’Haens G, Moja L, D’Hoore A, Panes J, Reinisch W, Sandborn WJ, Travis SP, Vermeire S, Peyrin-Biroulet L, Colombel JF. Development of red flags index for early referral of adults with symptoms and signs suggestive of Crohn’s disease: an IOIBD initiative. J Crohns Colitis. 2015;9:601–6. 25908718 10.1093/ecco-jcc/jjv067

[CR38] Dignass A, Van Assche G, Lindsay JO, Lemann M, Soderholm J, Colombel JF, Danese S, D’Hoore A, Gassull M, Gomollon F, Hommes DW, Michetti P, O’Morain C, Oresland T, Windsor A, Stange EF, Travis SP, European, C.s. and Colitis, O. The second European evidence-based Consensus on the diagnosis and management of Crohn’s disease: current management. J Crohns Colitis. 2010;4:28–62. 21122489 10.1016/j.crohns.2009.12.002

[CR39] Dikic I, Elazar Z. Mechanism and medical implications of mammalian autophagy. Nat Rev Mol Cell Biol. 2018;19:349–64. 29618831 10.1038/s41580-018-0003-4

[CR40] Djuric Z, Saranac L, Budic I, Pavlovic V, Djordjevic J. Therapeutic role of methotrexate in pediatric Crohn’s disease. Bosn J Basic Med Sci. 2018;18:211–6. 29338679 10.17305/bjbms.2018.2792 PMC6087553

[CR41] Eglinton TW, Barclay ML, Gearry RB, Frizelle FA. The spectrum of perianal Crohn’s disease in a population-based cohort. Dis Colon Rectum. 2012;55:773–7. 22706129 10.1097/DCR.0b013e31825228b0

[CR42] Fernandez AF, Lopez-Otin C. The functional and pathologic relevance of autophagy proteases. J Clin Invest. 2015;125:33–41. 25654548 10.1172/JCI73940 PMC4382236

[CR43] Feuerstein JD, Cheifetz AS. Crohn disease: epidemiology, diagnosis, and management. Mayo Clin Proc. 2017;92:1088–103. 28601423 10.1016/j.mayocp.2017.04.010

[CR44] Franke A, McGovern DP, Barrett JC, Wang K, Radford-Smith GL, Ahmad T, Lees CW, Balschun T, Lee J, Roberts R, Anderson CA, Bis JC, Bumpstead S, Ellinghaus D, Festen EM, Georges M, Green T, Haritunians T, Jostins L, Latiano A, Mathew CG, Montgomery GW, Prescott NJ, Raychaudhuri S, Rotter JI, Schumm P, Sharma Y, Simms LA, Taylor KD, Whiteman D, Wijmenga C, Baldassano RN, Barclay M, Bayless TM, Brand S, Buning C, Cohen A, Colombel JF, Cottone M, Stronati L, Denson T, De Vos M, D’Inca R, Dubinsky M, Edwards C, Florin T, Franchimont D, Gearry R, Glas J, Van Gossum A, Guthery SL, Halfvarson J, Verspaget HW, Hugot JP, Karban A, Laukens D, Lawrance I, Lemann M, Levine A, Libioulle C, Louis E, Mowat C, Newman W, Panes J, Phillips A, Proctor DD, Regueiro M, Russell R, Rutgeerts P, Sanderson J, Sans M, Seibold F, Steinhart AH, Stokkers PC, Torkvist L, Kullak-Ublick G, Wilson D, Walters T, Targan SR, Brant SR, Rioux JD, D’Amato M, Weersma RK, Kugathasan S, Griffiths AM, Mansfield JC, Vermeire S, Duerr RH, Silverberg MS, Satsangi J, Schreiber S, Cho JH, Annese V, Hakonarson H, Daly MJ, Parkes M. Genome-wide meta-analysis increases to 71 the number of confirmed Crohn’s disease susceptibility loci. Nat Genet. 2010;42:1118–25. 21102463 10.1038/ng.717 PMC3299551

[CR45] Fridh V, Rittinger K. The tandem CARDs of NOD2: intramolecular interactions and recognition of RIP2. PLoS ONE. 2012;7:e34375. 22470564 10.1371/journal.pone.0034375 PMC3314614

[CR46] Fujita N, Itoh T, Omori H, Fukuda M, Noda T, Yoshimori T. The Atg16L complex specifies the site of LC3 lipidation for membrane biogenesis in autophagy. Mol Biol Cell. 2008;19:2092–100. 18321988 10.1091/mbc.E07-12-1257 PMC2366860

[CR47] Ganley IG, du Lam H, Wang J, Ding X, Chen S, Jiang X. ULK1.ATG13.FIP200 complex mediates mTOR signaling and is essential for autophagy. J Biol Chem. 2009;284:12297–305. 19258318 10.1074/jbc.M900573200 PMC2673298

[CR48] Gao Y, Lu LJ, Zhang ZZ, Yang X, Du J, Wen K, Huang H, Wang XP, Sun XL. Xue-Jie-San prevents the early development of colitis-associated intestinal fibrosis by blocking Notch1 and FGL1 signaling pathways. J Ethnopharmacol. 2023;315:116678. 37263315 10.1016/j.jep.2023.116678

[CR49] Garrett WS, Gordon JI, Glimcher LH. Homeostasis and inflammation in the intestine. Cell. 2010;140:859–70. 20303876 10.1016/j.cell.2010.01.023 PMC2845719

[CR50] Glick D, Barth S, Macleod KF. Autophagy: cellular and molecular mechanisms. J Pathol. 2010;221:3–12. 20225336 10.1002/path.2697 PMC2990190

[CR51] Grimes CL, Ariyananda Lde Z, Melnyk JE, O’Shea EK. The innate immune protein Nod2 binds directly to MDP, a bacterial cell wall fragment. J Am Chem Soc. 2012;134:13535–7. 22857257 10.1021/ja303883c PMC3424850

[CR52] Guo X, Zhang W, Wang C, Zhang B, Li R, Zhang L, Zhao K, Li Y, Tian L, Li B, Cheng H, Li L, Pei C, Xu H. IRGM promotes the PINK1-mediated mitophagy through the degradation of Mitofilin in SH-SY5Y cells. FASEB J. 2020;34:14768–79. 32939830 10.1096/fj.202000943RR

[CR53] Hampe J, Franke A, Rosenstiel P, Till A, Teuber M, Huse K, Albrecht M, Mayr G, De La Vega FM, Briggs J, Gunther S, Prescott NJ, Onnie CM, Hasler R, Sipos B, Folsch UR, Lengauer T, Platzer M, Mathew CG, Krawczak M, Schreiber S. A genome-wide association scan of nonsynonymous SNPs identifies a susceptibility variant for Crohn disease in ATG16L1. Nat Genet. 2007;39:207–11. 17200669 10.1038/ng1954

[CR54] Haq S, Wang H, Grondin J, Banskota S, Marshall JK, Khan II, Chauhan U, Cote F, Kwon YH, Philpott D, Brumell JH, Surette M, Steinberg GR, Khan WI. Disruption of autophagy by increased 5-HT alters gut microbiota and enhances susceptibility to experimental colitis and Crohn’s disease. Sci Adv. 2021;7:6442. 10.1126/sciadv.abi6442 PMC8570609 34739317

[CR55] Hasegawa M, Fujimoto Y, Lucas PC, Nakano H, Fukase K, Nunez G, Inohara N. A critical role of RICK/RIP2 polyubiquitination in Nod-induced NF-kappaB activation. EMBO J. 2008;27:373–83. 18079694 10.1038/sj.emboj.7601962 PMC2234345

[CR56] He Y, Hara H, Nunez G. Mechanism and Regulation of NLRP3 Inflammasome Activation. Trends Biochem Sci. 2016;41:1012–21. 27669650 10.1016/j.tibs.2016.09.002 PMC5123939

[CR57] Hemshekhar M, Anaparti V, Mookherjee N. Functions of Cationic Host Defense Peptides in Immunity. Pharmaceuticals (basel). 2016;9(3):40. 27384571 10.3390/ph9030040 PMC5039493

[CR58] Henckaerts L, Cleynen I, Brinar M, John JM, Van Steen K, Rutgeerts P, Vermeire S. Genetic variation in the autophagy gene ULK1 and risk of Crohn’s disease. Inflamm Bowel Dis. 2011;17:1392–7. 21560199 10.1002/ibd.21486

[CR59] Hinterleitner TA, Petritsch W, Aichbichler B, Fickert P, Ranner G, Krejs GJ. Combination of cyclosporine, azathioprine and prednisolone for perianal fistulas in Crohn’s disease. Z Gastroenterol. 1997;35:603–8. 9297775

[CR60] Ho J, Zhang L, Liu X, Wong SH, Wang MHT, Lau BWM, Ngai SPC, Chan H, Choi G, Leung CCH, Wong WT, Tsang S, Gin T, Yu J, Chan MTV, Wu WKK. Pathological Role and Diagnostic Value of Endogenous Host Defense Peptides in Adult and Neonatal Sepsis: A Systematic Review. Shock. 2017;47:673–9. 27941592 10.1097/SHK.0000000000000815

[CR61] Holvoet T, Devriese S, Castermans K, Boland S, Leysen D, Vandewynckel YP, Devisscher L, Van den Bossche L, Van Welden S, Dullaers M, Vandenbroucke RE, De Rycke R, Geboes K, Bourin A, Defert O, Hindryckx P, De Vos M, Laukens D. Treatment of Intestinal Fibrosis in Experimental Inflammatory Bowel Disease by the Pleiotropic Actions of a Local Rho Kinase Inhibitor. Gastroenterology. 2017;153:1054–67. 28642198 10.1053/j.gastro.2017.06.013

[CR62] Homer CR, Richmond AL, Rebert NA, Achkar JP, McDonald C. ATG16L1 and NOD2 interact in an autophagy-dependent antibacterial pathway implicated in Crohn’s disease pathogenesis. Gastroenterology. 2010;139:1630–41, 1641 e1-2. 20637199 10.1053/j.gastro.2010.07.006 PMC2967588

[CR63] Honjo H, Watanabe T, Arai Y, Kamata K, Minaga K, Komeda Y, Yamashita K, Kudo M. ATG16L1 negatively regulates RICK/RIP2-mediated innate immune responses. Int Immunol. 2021;33:91–105. 32909611 10.1093/intimm/dxaa062

[CR64] Hooper KM, Barlow PG, Stevens C, Henderson P. Inflammatory bowel disease drugs: a focus on autophagy. J Crohns Colitis. 2017;11:118–27. 27381462 10.1093/ecco-jcc/jjw127 PMC5175491

[CR65] Hugot JP, Chamaillard M, Zouali H, Lesage S, Cezard JP, Belaiche J, Almer S, Tysk C, O’Morain CA, Gassull M, Binder V, Finkel Y, Cortot A, Modigliani R, Laurent-Puig P, Gower-Rousseau C, Macry J, Colombel JF, Sahbatou M, Thomas G. Association of NOD2 leucine-rich repeat variants with susceptibility to Crohn’s disease. Nature. 2001;411:599–603. 11385576 10.1038/35079107

[CR66] Iorio R, Celenza G, Petricca S. Mitophagy: molecular mechanisms, new concepts on parkin activation and the emerging role of AMPK/ULK1 Axis. Cells. 2021;11:30. 35011593 10.3390/cells11010030 PMC8750607

[CR67] Itakura E, Mizushima N. Characterization of autophagosome formation site by a hierarchical analysis of mammalian Atg proteins. Autophagy. 2010;6:764–76. 20639694 10.4161/auto.6.6.12709 PMC3321844

[CR68] Jang BY, Guo SB, Bai R, Liu WL, Gong YL, Zhao ZQ. Methylprednisolone Inhibits Autophagy of Vascular Endothelial Cells in Rat Femoral Head Via PI3K/Akt/mTOR Pathway. Orthop Surg. 2022;14:2669–81. 36052745 10.1111/os.13369 PMC9531065

[CR69] Jiang W, Chen X, Ji C, Zhang W, Song J, Li J, Wang J. Key regulators of autophagosome closure. Cells. 2021;10:2814. 34831036 10.3390/cells10112814 PMC8616111

[CR70] Ke P, Shao BZ, Xu ZQ, Wei W, Han BZ, Chen XW, Su DF, Liu C. Activation of Cannabinoid Receptor 2 Ameliorates DSS-Induced Colitis through Inhibiting NLRP3 Inflammasome in Macrophages. PLoS ONE. 2016;11:e0155076. 27611972 10.1371/journal.pone.0155076 PMC5017608

[CR71] Kim J, Kundu M, Viollet B, Guan KL. AMPK and mTOR regulate autophagy through direct phosphorylation of Ulk1. Nat Cell Biol. 2011;13:132–41. 21258367 10.1038/ncb2152 PMC3987946

[CR72] Klionsky DJ, Petroni G, Amaravadi RK, Baehrecke EH, Ballabio A, Boya P, Bravo-San Pedro JM, Cadwell K, Cecconi F, Choi AMK, Choi ME, Chu CT, Codogno P, Colombo MI, Cuervo AM, Deretic V, Dikic I, Elazar Z, Eskelinen EL, Fimia GM, Gewirtz DA, Green DR, Hansen M, Jaattela M, Johansen T, Juhasz G, Karantza V, Kraft C, Kroemer G, Ktistakis NT, Kumar S, Lopez-Otin C, Macleod KF, Madeo F, Martinez J, Melendez A, Mizushima N, Munz C, Penninger JM, Perera RM, Piacentini M, Reggiori F, Rubinsztein DC, Ryan KM, Sadoshima J, Santambrogio L, Scorrano L, Simon HU, Simon AK, Simonsen A, Stolz A, Tavernarakis N, Tooze SA, Yoshimori T, Yuan J, Yue Z, Zhong Q, Galluzzi L, Pietrocola F. Autophagy in major human diseases. EMBO J. 2021;40:e108863. 34459017 10.15252/embj.2021108863 PMC8488577

[CR73] Krieg A, Correa RG, Garrison JB, Le Negrate G, Welsh K, Huang Z, Knoefel WT, Reed JC. XIAP mediates NOD signaling via interaction with RIP2. Proc Natl Acad Sci U S A. 2009;106:14524–9. 19667203 10.1073/pnas.0907131106 PMC2732880

[CR74] Kuma A, Mizushima N, Ishihara N, Ohsumi Y. Formation of the approximately 350-kDa Apg12-Apg5.Apg16 multimeric complex, mediated by Apg16 oligomerization, is essential for autophagy in yeast. J Biol Chem. 2002;277:18619–25. 11897782 10.1074/jbc.M111889200

[CR75] Kumar S, Jain A, Farzam F, Jia J, Gu Y, Choi SW, Mudd MH, Claude-Taupin A, Wester MJ, Lidke KA, Rusten TE, Deretic V. Mechanism of Stx17 recruitment to autophagosomes via IRGM and mammalian Atg8 proteins. J Cell Biol. 2018;217:997–1013. 29420192 10.1083/jcb.201708039 PMC5839791

[CR76] Kumar S, Jain A, Choi SW, da Silva GPD, Allers L, Mudd MH, Peters RS, Anonsen JH, Rusten TE, Lazarou M, Deretic V. Mammalian Atg8 proteins and the autophagy factor IRGM control mTOR and TFEB at a regulatory node critical for responses to pathogens. Nat Cell Biol. 2020;22:973–85. 32753672 10.1038/s41556-020-0549-1 PMC7482486

[CR77] Kuo SY, Castoreno AB, Aldrich LN, Lassen KG, Goel G, Dancik V, Kuballa P, Latorre I, Conway KL, Sarkar S, Maetzel D, Jaenisch R, Clemons PA, Schreiber SL, Shamji AF, Xavier RJ. Small-molecule enhancers of autophagy modulate cellular disease phenotypes suggested by human genetics. Proc Natl Acad Sci U S A. 2015;112:E4281–7. 26195741 10.1073/pnas.1512289112 PMC4534235

[CR78] Lahiri A, Hedl M, Abraham C. MTMR3 risk allele enhances innate receptor-induced signaling and cytokines by decreasing autophagy and increasing caspase-1 activation. Proc Natl Acad Sci U S A. 2015;112:10461–6. 26240347 10.1073/pnas.1501752112 PMC4547281

[CR79] Lamb CA, Yoshimori T, Tooze SA. The autophagosome: origins unknown, biogenesis complex. Nat Rev Mol Cell Biol. 2013;14:759–74. 24201109 10.1038/nrm3696

[CR80] Lapaquette P, Glasser AL, Huett A, Xavier RJ, Darfeuille-Michaud A. Crohn’s disease-associated adherent-invasive E. coli are selectively favoured by impaired autophagy to replicate intracellularly. Cell Microbiol. 2010;12:99–113. 19747213 10.1111/j.1462-5822.2009.01381.x PMC3743084

[CR81] Lapaquette P, Brest P, Hofman P, Darfeuille-Michaud A. Etiology of Crohn’s disease: many roads lead to autophagy. J Mol Med (berl). 2012a;90:987–96. 22797958 10.1007/s00109-012-0934-8

[CR82] Lapaquette P, Bringer MA, Darfeuille-Michaud A. Defects in autophagy favour adherent-invasive Escherichia coli persistence within macrophages leading to increased pro-inflammatory response. Cell Microbiol. 2012b;14:791–807. 22309232 10.1111/j.1462-5822.2012.01768.x

[CR83] Lazarou M, Sliter DA, Kane LA, Sarraf SA, Wang C, Burman JL, Sideris DP, Fogel AI, Youle RJ. The ubiquitin kinase PINK1 recruits autophagy receptors to induce mitophagy. Nature. 2015;524:309–14. 26266977 10.1038/nature14893 PMC5018156

[CR84] Lin XT, Zheng XB, Fan DJ, Yao QQ, Hu JC, Lian L, Wu XJ, Lan P, He XS. MicroRNA-143 targets ATG2B to inhibit autophagy and increase inflammatory responses in Crohn’s disease. Inflamm Bowel Dis. 2018;24:781–91. 29562274 10.1093/ibd/izx075

[CR85] Liu Z, Lee J, Krummey S, Lu W, Cai H, Lenardo MJ. The kinase LRRK2 is a regulator of the transcription factor NFAT that modulates the severity of inflammatory bowel disease. Nat Immunol. 2011;12:1063–70. 21983832 10.1038/ni.2113 PMC4140245

[CR86] Liu TC, Naito T, Liu Z, VanDussen KL, Haritunians T, Li D, Endo K, Kawai Y, Nagasaki M, Kinouchi Y, McGovern DP, Shimosegawa T, Kakuta Y, Stappenbeck TS. LRRK2 but not ATG16L1 is associated with Paneth cell defect in Japanese Crohn’s disease patients. JCI Insight. 2017;2:e91917. 28352666 10.1172/jci.insight.91917 PMC5358495

[CR87] Lu C, Chen J, Xu HG, Zhou X, He Q, Li YL, Jiang G, Shan Y, Xue B, Zhao RX, Wang Y, Werle KD, Cui R, Liang J, Xu ZX. MIR106B and MIR93 prevent removal of bacteria from epithelial cells by disrupting ATG16L1-mediated autophagy. Gastroenterology. 2014;146:188–99. 24036151 10.1053/j.gastro.2013.09.006 PMC3870037

[CR88] Lueschow SR, McElroy SJ. The Paneth cell: the curator and defender of the immature small intestine. Front Immunol. 2020;11:587. 32308658 10.3389/fimmu.2020.00587 PMC7145889

[CR89] Ma C, Ascoytia C, McCarrier KP, Martin M, Feagan BG, Jairath V. Physicians’ perspectives on cost, safety, and perceived efficacy determine aminosalicylate use in Crohn’s disease. Dig Dis Sci. 2018;63:2555–63. 29959726 10.1007/s10620-018-5181-6

[CR90] Maeda S, Hsu LC, Liu H, Bankston LA, Iimura M, Kagnoff MF, Eckmann L, Karin M. Nod2 mutation in Crohn’s disease potentiates NF-kappaB activity and IL-1beta processing. Science. 2005;307:734–8. 15692052 10.1126/science.1103685

[CR91] Magro F, Cordeiro G, Dias AM, Estevinho MM. Inflammatory bowel disease - non-biological treatment. Pharmacol Res. 2020;160:105075. 32653651 10.1016/j.phrs.2020.105075

[CR92] Mahid SS, Minor KS, Soto RE, Hornung CA, Galandiuk S. Smoking and inflammatory bowel disease: a meta-analysis. Mayo Clin Proc. 2006;81:1462–71. 17120402 10.4065/81.11.1462

[CR93] Malchow H, Ewe K, Brandes JW, Goebell H, Ehms H, Sommer H, Jesdinsky H. European Cooperative Crohn’s disease Study (ECCDS): results of drug treatment. Gastroenterology. 1984;86:249–66. 6140202

[CR94] Mancias JD, Kimmelman AC. Mechanisms of selective autophagy in normal physiology and cancer. J Mol Biol. 2016;428:1659–80. 26953261 10.1016/j.jmb.2016.02.027 PMC4846542

[CR95] Maneechotesuwan K, Kasetsinsombat K, Wongkajornsilp A, Barnes PJ. Role of autophagy in regulating interleukin-10 and the responses to corticosteroids and statins in asthma. Clin Exp Allergy. 2021;51:1553–65. 33423318 10.1111/cea.13825

[CR96] Manzoni C, Mamais A, Roosen DA, Dihanich S, Soutar MP, Plun-Favreau H, Bandopadhyay R, Hardy J, Tooze SA, Cookson MR, Lewis PA. mTOR independent regulation of macroautophagy by Leucine Rich Repeat Kinase 2 via Beclin-1. Sci Rep. 2016;6:35106. 27731364 10.1038/srep35106 PMC5059726

[CR97] Massey DC, Bredin F, Parkes M. Use of sirolimus (rapamycin) to treat refractory Crohn’s disease. Gut. 2008;57:1294–6. 18719139 10.1136/gut.2008.157297

[CR98] McCarroll SA, Huett A, Kuballa P, Chilewski SD, Landry A, Goyette P, Zody MC, Hall JL, Brant SR, Cho JH, Duerr RH, Silverberg MS, Taylor KD, Rioux JD, Altshuler D, Daly MJ, Xavier RJ. Deletion polymorphism upstream of IRGM associated with altered IRGM expression and Crohn’s disease. Nat Genet. 2008;40:1107–12. 19165925 10.1038/ng.215 PMC2731799

[CR99] McDonald JW, Wang Y, Tsoulis DJ, MacDonald JK, Feagan BG. Methotrexate for induction of remission in refractory Crohn’s disease. Cochrane Database Syst Rev. 2014;2014:CD003459. 25099640 10.1002/14651858.CD003459.pub4 PMC7154581

[CR100] Medina DL, Di Paola S, Peluso I, Armani A, De Stefani D, Venditti R, Montefusco S, Scotto-Rosato A, Prezioso C, Forrester A, Settembre C, Wang W, Gao Q, Xu H, Sandri M, Rizzuto R, De Matteis MA, Ballabio A. Lysosomal calcium signalling regulates autophagy through calcineurin and TFEB. Nat Cell Biol. 2015;17:288–99. 25720963 10.1038/ncb3114 PMC4801004

[CR101] Mirkov MU, Verstockt B, Cleynen I. Genetics of inflammatory bowel disease: beyond NOD2. Lancet Gastroenterol Hepatol. 2017;2:224–34. 28404137 10.1016/S2468-1253(16)30111-X

[CR102] Mizushima N. A brief history of autophagy from cell biology to physiology and disease. Nat Cell Biol. 2018;20:521–7. 29686264 10.1038/s41556-018-0092-5

[CR103] Mizushima N, Komatsu M. Autophagy: renovation of cells and tissues. Cell. 2011;147:728–41. 22078875 10.1016/j.cell.2011.10.026

[CR104] Morgan S, Hooper KM, Milne EM, Farquharson C, Stevens C, Staines KA. Azathioprine has a deleterious effect on the bone health of mice with DSS-Induced inflammatory bowel disease. Int J Mol Sci. 2019;20(23):6085. 31816823 10.3390/ijms20236085 PMC6929096

[CR105] Mukherjee T, Hovingh ES, Foerster EG, Abdel-Nour M, Philpott DJ, Girardin SE. NOD1 and NOD2 in inflammation, immunity and disease. Arch Biochem Biophys. 2019;670:69–81. 30578751 10.1016/j.abb.2018.12.022

[CR106] Muzes G, Tulassay Z, Sipos F. Interplay of autophagy and innate immunity in Crohn’s disease: a key immunobiologic feature. World J Gastroenterol. 2013;19:4447–54. 23901219 10.3748/wjg.v19.i28.4447 PMC3725368

[CR107] Nguyen HT, Lapaquette P, Bringer MA, Darfeuille-Michaud A. Autophagy and Crohn’s disease. J Innate Immun. 2013;5:434–43. 23328432 10.1159/000345129 PMC6741541

[CR108] Ni Z, Li H, Mu D, Hou J, Liu X, Tang S, Zheng S. Rapamycin alleviates 2,4,6-trinitrobenzene sulfonic acid-induced colitis through autophagy induction and NF-kappaB pathway inhibition in mice. Mediators Inflamm. 2022;2022:2923216. 36032781 10.1155/2022/2923216 PMC9410967

[CR109] Noah TK, Donahue B, Shroyer NF. Intestinal development and differentiation. Exp Cell Res. 2011;317:2702–10. 21978911 10.1016/j.yexcr.2011.09.006 PMC3210330

[CR110] Noda T. Autophagy in the context of the cellular membrane-trafficking system: the enigma of Atg9 vesicles. Biochem Soc Trans. 2017;45:1323–31. 29150528 10.1042/BST20170128 PMC5730941

[CR111] Noureldin M, Cohen-Mekelburg S, Mahmood A, Stidham R, Higgins PDR, Govani S, Deshpande AR, Waljee AK. Trends of 5-Aminosalicylate medication use in patients with Crohn disease. Inflamm Bowel Dis. 2021;27:516–21. 32469067 10.1093/ibd/izaa127 PMC8861365

[CR112] Nys K, Agostinis P, Vermeire S. Autophagy: a new target or an old strategy for the treatment of Crohn’s disease? Nat Rev Gastroenterol Hepatol. 2013;10:395–401. 23591407 10.1038/nrgastro.2013.66

[CR113] Ogura Y, Bonen DK, Inohara N, Nicolae DL, Chen FF, Ramos R, Britton H, Moran T, Karaliuskas R, Duerr RH, Achkar JP, Brant SR, Bayless TM, Kirschner BS, Hanauer SB, Nunez G, Cho JH. A frameshift mutation in NOD2 associated with susceptibility to Crohn’s disease. Nature. 2001;411:603–6. 11385577 10.1038/35079114

[CR114] Okai N, Watanabe T, Minaga K, Kamata K, Honjo H, Kudo M. Alterations of autophagic and innate immune responses by the Crohn’s disease-associated ATG16L1 mutation. World J Gastroenterol. 2022;28:3063–70. 36051337 10.3748/wjg.v28.i26.3063 PMC9331526

[CR115] Pallet N, Bouvier N, Legendre C, Gilleron J, Codogno P, Beaune P, Thervet E, Anglicheau D. Autophagy protects renal tubular cells against cyclosporine toxicity. Autophagy. 2008;4:783–91. 18628650 10.4161/auto.6477

[CR116] Palmela C, Chevarin C, Xu Z, Torres J, Sevrin G, Hirten R, Barnich N, Ng SC, Colombel JF. Adherent-invasive Escherichia coli in inflammatory bowel disease. Gut. 2018;67:574–87. 29141957 10.1136/gutjnl-2017-314903

[CR117] Parian AM, Obi M, Fleshner P, Schwartz DA. Management of Perianal Crohn’s disease. Am J Gastroenterol. 2023;118:1323–31. 37207318 10.14309/ajg.0000000000002326

[CR118] Parkes M, Barrett JC, Prescott NJ, Tremelling M, Anderson CA, Fisher SA, Roberts RG, Nimmo ER, Cummings FR, Soars D, Drummond H, Lees CW, Khawaja SA, Bagnall R, Burke DA, Todhunter CE, Ahmad T, Onnie CM, McArdle W, Strachan D, Bethel G, Bryan C, Lewis CM, Deloukas P, Forbes A, Sanderson J, Jewell DP, Satsangi J, Mansfield JC, Wellcome Trust Case Control, C, Cardon L, Mathew CG. Sequence variants in the autophagy gene IRGM and multiple other replicating loci contribute to Crohn’s disease susceptibility. Nat Genet. 2007;39:830–2. 17554261 10.1038/ng2061 PMC2628541

[CR119] Plaze M, Attali D, Prot M, Petit AC, Blatzer M, Vinckier F, Levillayer L, Chiaravalli J, Perin-Dureau F, Cachia A, Friedlander G, Chretien F, Simon-Loriere E, Gaillard R. Inhibition of the replication of SARS-CoV-2 in human cells by the FDA-approved drug chlorpromazine. Int J Antimicrob Agents. 2021;57:106274. 33387629 10.1016/j.ijantimicag.2020.106274 PMC7772996

[CR120] Prescott NJ, Dominy KM, Kubo M, Lewis CM, Fisher SA, Redon R, Huang N, Stranger BE, Blaszczyk K, Hudspith B, Parkes G, Hosono N, Yamazaki K, Onnie CM, Forbes A, Dermitzakis ET, Nakamura Y, Mansfield JC, Sanderson J, Hurles ME, Roberts RG, Mathew CG. Independent and population-specific association of risk variants at the IRGM locus with Crohn’s disease. Hum Mol Genet. 2010;19:1828–39. 20106866 10.1093/hmg/ddq041 PMC2850616

[CR121] Randhawa R, Duseja A, Changotra H. A novel Tetra-primer ARMS-PCR based assay for genotyping SNP rs12303764(G/T) of human Unc-51 like kinase 1 gene. Mol Biol Rep. 2017;44:1–4. 27783190 10.1007/s11033-016-4087-7

[CR122] Ridler C. Parkinson disease: LRRK2 variants linked to PD and Crohn’s disease. Nat Rev Neurol. 2018;14:126. 29391585 10.1038/nrneurol.2018.10

[CR123] Rioux JD, Xavier RJ, Taylor KD, Silverberg MS, Goyette P, Huett A, Green T, Kuballa P, Barmada MM, Datta LW, Shugart YY, Griffiths AM, Targan SR, Ippoliti AF, Bernard EJ, Mei L, Nicolae DL, Regueiro M, Schumm LP, Steinhart AH, Rotter JI, Duerr RH, Cho JH, Daly MJ, Brant SR. Genome-wide association study identifies new susceptibility loci for Crohn disease and implicates autophagy in disease pathogenesis. Nat Genet. 2007;39:596–604. 17435756 10.1038/ng2032 PMC2757939

[CR124] Rizzo V, Ferlazzo N, Curro M, Isola G, Matarese M, Bertuccio MP, Caccamo D, Matarese G, Ientile R. Baicalin-induced autophagy preserved LPS-Stimulated intestinal cells from inflammation and alterations of paracellular permeability. Int J Mol Sci. 2021;22:2315. 33652555 10.3390/ijms22052315 PMC7956379

[CR125] Roda G, Chien Ng S, Kotze PG, Argollo M, Panaccione R, Spinelli A, Kaser A, Peyrin-Biroulet L, Danese S. Crohn’s Disease. Nat Rev Dis Primers. 2020;6:22. 32242028 10.1038/s41572-020-0156-2

[CR126] Ruthruff B. Clinical review of Crohn’s disease. J Am Acad Nurse Pract. 2007;19:392–7. 17655568 10.1111/j.1745-7599.2007.00242.x

[CR127] Sabers CJ, Martin MM, Brunn GJ, Williams JM, Dumont FJ, Wiederrecht G, Abraham RT. Isolation of a protein target of the FKBP12-rapamycin complex in mammalian cells. J Biol Chem. 1995;270:815–22. 7822316 10.1074/jbc.270.2.815

[CR128] Saitoh T, Fujita N, Jang MH, Uematsu S, Yang BG, Satoh T, Omori H, Noda T, Yamamoto N, Komatsu M, Tanaka K, Kawai T, Tsujimura T, Takeuchi O, Yoshimori T, Akira S. Loss of the autophagy protein Atg16L1 enhances endotoxin-induced IL-1beta production. Nature. 2008;456:264–8. 18849965 10.1038/nature07383

[CR129] Samie M, Lim J, Verschueren E, Baughman JM, Peng I, Wong A, Kwon Y, Senbabaoglu Y, Hackney JA, Keir M, McKenzie B, Kirkpatrick DS, van Lookeren Campagne M, Murthy A. Selective autophagy of the adaptor TRIF regulates innate inflammatory signaling. Nat Immunol. 2018;19:246–54. 29358708 10.1038/s41590-017-0042-6

[CR130] Sandborn WJ, Feagan BG, Lichtenstein GR. Medical management of mild to moderate Crohn’s disease: evidence-based treatment algorithms for induction and maintenance of remission. Aliment Pharmacol Ther. 2007;26:987–1003. 17877506 10.1111/j.1365-2036.2007.03455.x

[CR131] Sasson AN, Ananthakrishnan AN, Raman M. Diet in treatment of inflammatory bowel diseases. Clin Gastroenterol Hepatol. 2021;19:425-435 e3. 31812656 10.1016/j.cgh.2019.11.054

[CR132] Savelkoul EHJ, Maas MHJ, Bourgonje AR, Crouwel F, Biemans VBC, den Broeder N, Russel M, Romkens TEH, de Boer NK, Dijkstra G, Hoentjen F. Favourable tolerability and drug survival of tioguanine versus methotrexate after failure of conventional thiopurines in Crohn’s disease. J Crohns Colitis. 2022;16:1372–9. 35303065 10.1093/ecco-jcc/jjac044 PMC9455785

[CR133] Sazonovs A, Stevens CR, Venkataraman GR, Yuan K, Avila B, Abreu MT, Ahmad T, Allez M, Ananthakrishnan AN, Atzmon G, Baras A, Barrett JC, Barzilai N, Beaugerie L, Beecham A, Bernstein CN, Bitton A, Bokemeyer B, Chan A, Chung D, Cleynen I, Cosnes J, Cutler DJ, Daly A, Damas OM, Datta LW, Dawany N, Devoto M, Dodge S, Ellinghaus E, Fachal L, Farkkila M, Faubion W, Ferreira M, Franchimont D, Gabriel SB, Ge T, Georges M, Gettler K, Giri M, Glaser B, Goerg S, Goyette P, Graham D, Hamalainen E, Haritunians T, Heap GA, Hiltunen M, Hoeppner M, Horowitz JE, Irving P, Iyer V, Jalas C, Kelsen J, Khalili H, Kirschner BS, Kontula K, Koskela JT, Kugathasan S, Kupcinskas J, Lamb CA, Laudes M, Levesque C, Levine AP, Lewis JD, Liefferinckx C, Loescher BS, Louis E, Mansfield J, May S, McCauley JL, Mengesha E, Mni M, Moayyedi P, Moran CJ, Newberry RD, O’Charoen S, Okou DT, Oldenburg B, Ostrer H, Palotie A, Paquette J, Pekow J, Peter I, Pierik MJ, Ponsioen CY, Pontikos N, Prescott N, Pulver AE, Rahmouni S, Rice DL, Saavalainen P, Sands B, Sartor RB, Schiff ER, Schreiber S, Schumm LP, Segal AW, Seksik P, Shawky R, et al. Large-scale sequencing identifies multiple genes and rare variants associated with Crohn’s disease susceptibility. Nat Genet. 2022;54:1275–83. 36038634 10.1038/s41588-022-01156-2 PMC9700438

[CR134] Scharl M, Mwinyi J, Fischbeck A, Leucht K, Eloranta JJ, Arikkat J, Pesch T, Kellermeier S, Mair A, Kullak-Ublick GA, Truninger K, Noreen F, Regula J, Gaj P, Pittet V, Mueller C, Hofmann C, Fried M, McCole DF, Rogler G. Crohn’s disease-associated polymorphism within the PTPN2 gene affects muramyl-dipeptide-induced cytokine secretion and autophagy. Inflamm Bowel Dis. 2012a;18:900–12. 22021207 10.1002/ibd.21913

[CR135] Scharl M, Wojtal KA, Becker HM, Fischbeck A, Frei P, Arikkat J, Pesch T, Kellermeier S, Boone DL, Weber A, Loessner MJ, Vavricka SR, Fried M, McCole DF, Rogler G. Protein tyrosine phosphatase nonreceptor type 2 regulates autophagosome formation in human intestinal cells. Inflamm Bowel Dis. 2012b;18:1287–302. 21987459 10.1002/ibd.21891

[CR136] Schwerd T, Pandey S, Yang HT, Bagola K, Jameson E, Jung J, Lachmann RH, Shah N, Patel SY, Booth C, Runz H, Duker G, Bettels R, Rohrbach M, Kugathasan S, Chapel H, Keshav S, Elkadri A, Platt N, Muise AM, Koletzko S, Xavier RJ, Marquardt T, Powrie F, Wraith JE, Gyrd-Hansen M, Platt FM, Uhlig HH. Impaired antibacterial autophagy links granulomatous intestinal inflammation in Niemann-Pick disease type C1 and XIAP deficiency with NOD2 variants in Crohn’s disease. Gut. 2017;66:1060–73. 26953272 10.1136/gutjnl-2015-310382 PMC5532464

[CR137] Shima T, Kirisako H, Nakatogawa H. COPII vesicles contribute to autophagosomal membranes. J Cell Biol. 2019;218:1503–10. 30787039 10.1083/jcb.201809032 PMC6504894

[CR138] Song J, Lan J, Tang J, Luo N. PTPN2 in the immunity and tumor immunotherapy: a concise review. Int J Mol Sci. 2022;23(17):10025. 36077422 10.3390/ijms231710025 PMC9456094

[CR139] Stafford CA, Gassauer AM, de Oliveira Mann CC, Tanzer MC, Fessler E, Wefers B, Nagl D, Kuut G, Sulek K, Vasilopoulou C, Schwojer SJ, Wiest A, Pfautsch MK, Wurst W, Yabal M, Frohlich T, Mann M, Gisch N, Jae LT, Hornung V. Phosphorylation of muramyl peptides by NAGK is required for NOD2 activation. Nature. 2022;609:590–6. 36002575 10.1038/s41586-022-05125-x PMC9477735

[CR140] Strober W, Fuss IJ. Proinflammatory cytokines in the pathogenesis of inflammatory bowel diseases. Gastroenterology. 2011;140:1756–67. 21530742 10.1053/j.gastro.2011.02.016 PMC3773507

[CR141] Taguchi-Atarashi N, Hamasaki M, Matsunaga K, Omori H, Ktistakis NT, Yoshimori T, Noda T. Modulation of local PtdIns3P levels by the PI phosphatase MTMR3 regulates constitutive autophagy. Traffic. 2010;11:468–78. 20059746 10.1111/j.1600-0854.2010.01034.x

[CR142] Takagawa T, Kitani A, Fuss I, Levine B, Brant SR, Peter I, Tajima M, Nakamura S, Strober W. An increase in LRRK2 suppresses autophagy and enhances Dectin-1-induced immunity in a mouse model of colitis. Sci Transl Med. 2018;10:eaan8162. 29875204 10.1126/scitranslmed.aan8162 PMC6636639

[CR143] Takeuchi O, Akira S. Pattern recognition receptors and inflammation. Cell. 2010;140:805–20. 20303872 10.1016/j.cell.2010.01.022

[CR144] Taylor GA. IRG proteins: key mediators of interferon-regulated host resistance to intracellular pathogens. Cell Microbiol. 2007;9:1099–107. 17359233 10.1111/j.1462-5822.2007.00916.x

[CR145] Torres J, Mehandru S, Colombel JF, Peyrin-Biroulet L. Crohn’s disease. Lancet. 2017;389:1741–55. 27914655 10.1016/S0140-6736(16)31711-1

[CR146] Travassos LH, Carneiro LA, Ramjeet M, Hussey S, Kim YG, Magalhaes JG, Yuan L, Soares F, Chea E, Le Bourhis L, Boneca IG, Allaoui A, Jones NL, Nunez G, Girardin SE, Philpott DJ. Nod1 and Nod2 direct autophagy by recruiting ATG16L1 to the plasma membrane at the site of bacterial entry. Nat Immunol. 2010;11:55–62. 19898471 10.1038/ni.1823

[CR147] Ulm H, Wilmes M, Shai Y, Sahl HG. Antimicrobial host defensins - specific antibiotic activities and innate defense modulation. Front Immunol. 2012;3:249. 22912635 10.3389/fimmu.2012.00249 PMC3418506

[CR148] Ungaro R, Bernstein CN, Gearry R, Hviid A, Kolho KL, Kronman MP, Shaw S, Van Kruiningen H, Colombel JF, Atreja A. Antibiotics associated with increased risk of new-onset Crohn’s disease but not ulcerative colitis: a meta-analysis. Am J Gastroenterol. 2014;109:1728–38. 25223575 10.1038/ajg.2014.246

[CR149] Ungaro R, Chang HL, Cote-Daigneault J, Mehandru S, Atreja A, Colombel JF. Statins associated with decreased risk of new onset inflammatory bowel disease. Am J Gastroenterol. 2016;111:1416–23. 27296939 10.1038/ajg.2016.233

[CR150] Uskudar Cansu D, Bodakci E, Korkmaz C. Dose-dependent bradycardia as a rare side effect of corticosteroids: a case report and review of the literature. Rheumatol Int. 2018;38:2337–43. 30276424 10.1007/s00296-018-4167-1

[CR151] Van Limbergen J, Wilson DC, Satsangi J. The genetics of Crohn’s disease. Annu Rev Genomics Hum Genet. 2009;10:89–116. 19453248 10.1146/annurev-genom-082908-150013

[CR152] Vavricka SR, Brun L, Ballabeni P, Pittet V, Prinz Vavricka BM, Zeitz J, Rogler G, Schoepfer AM. Frequency and risk factors for extraintestinal manifestations in the Swiss inflammatory bowel disease cohort. Am J Gastroenterol. 2011;106:110–9. 20808297 10.1038/ajg.2010.343

[CR153] Vavricka SR, Schoepfer AM, Scharl M, Rogler G. Steroid use in Crohn’s disease. Drugs. 2014;74:313–24. 24532122 10.1007/s40265-014-0183-y

[CR154] Venner JM, Bernstein CN. Immunomodulators: still having a role? Gastroenterol Rep (oxf). 2022;10:goac061. 36381225 10.1093/gastro/goac061 PMC9642324

[CR155] Vergne I, Deretic V. The role of PI3P phosphatases in the regulation of autophagy. FEBS Lett. 2010;584:1313–8. 20188094 10.1016/j.febslet.2010.02.054 PMC2885894

[CR156] Verstockt B, Smith KG, Lee JC. Genome-wide association studies in Crohn’s disease: past, present and future. Clin Transl Immunology. 2018;7:e1001. 29484179 10.1002/cti2.1001 PMC5822399

[CR157] Watanabe T, Asano N, Murray PJ, Ozato K, Tailor P, Fuss IJ, Kitani A, Strober W. Muramyl dipeptide activation of nucleotide-binding oligomerization domain 2 protects mice from experimental colitis. J Clin Invest. 2008;118:545–59. 18188453 10.1172/JCI33145 PMC2176188

[CR158] Windheim M, Lang C, Peggie M, Plater LA, Cohen P. Molecular mechanisms involved in the regulation of cytokine production by muramyl dipeptide. Biochem J. 2007;404:179–90. 17348859 10.1042/BJ20061704 PMC1868792

[CR159] Xiong S, Song D, Xiang Y, Li Y, Zhong Y, Li H, Zhang P, Zhou W, Zeng X, Zhang X. Reactive oxygen species, not Ca(2+), mediates methotrexate-induced autophagy and apoptosis in spermatocyte cell line. Basic Clin Pharmacol Toxicol. 2020;126:144–52. 31420979 10.1111/bcpt.13306

[CR160] Xu YR, Lei CQ. TAK1-TABs complex: a central signalosome in inflammatory responses. Front Immunol. 2020;11:608976. 33469458 10.3389/fimmu.2020.608976 PMC7813674

[CR161] Xu XS, Shao N, Duan XT, Zhang X, Zhang YF. Tacrolimus alleviates Ox-LDL damage through inducing vascular endothelial autophagy. Eur Rev Med Pharmacol Sci. 2018;22:3199–206. 29863266 10.26355/eurrev_201805_15081

[CR162] Yan R, Liu Z. LRRK2 enhances Nod1/2-mediated inflammatory cytokine production by promoting Rip2 phosphorylation. Protein Cell. 2017;8:55–66. 27830463 10.1007/s13238-016-0326-x PMC5233611

[CR163] Yang YX, Lichtenstein GR. Corticosteroids in Crohn’s disease. Am J Gastroenterol. 2002;97:803–23. 12003413 10.1111/j.1572-0241.2002.05596.x

[CR164] Yang XQ, Yu SY, Yu L, Ge L, Zhang Y, Hao ZH, Liu GS. Effects of tacrolimus on autophagy protein LC3 in puromycin-damaged mouse podocytes. J Int Med Res. 2020;48:300060520971422. 33322998 10.1177/0300060520971422 PMC7745617

[CR165] Yasueda A, Kayama H, Murohashi M, Nishimura J, Wakame K, Komatsu KI, Ogino T, Miyoshi N, Takahashi H, Uemura M, Matsuda C, Kitagawa T, Takeda K, Ito T, Doki Y, Eguchi H, Shimizu S, Mizushima T. Sanguisorba officinalis L. derived from herbal medicine prevents intestinal inflammation by inducing autophagy in macrophages. Sci Rep. 2020;10:9972. 32561763 10.1038/s41598-020-65306-4 PMC7305163

[CR166] Zachari M, Ganley IG. The mammalian ULK1 complex and autophagy initiation. Essays Biochem. 2017;61:585–96. 29233870 10.1042/EBC20170021 PMC5869855

[CR167] Zhang L, Liu B. Targeting autophagy with small-molecule modulators in immune-related diseases. Adv Exp Med Biol. 2019;1209:181–203. 31728871 10.1007/978-981-15-0606-2_11

[CR168] Zhang W, Yan C, Xiao Y, Sun Y, Lin Y, Li Q, Cai W. Sulfasalazine induces autophagy inhibiting neointimal hyperplasia following carotid artery injuries in mice. Front Bioeng Biotechnol. 2023;11:1199785. 37288359 10.3389/fbioe.2023.1199785 PMC10242098

[CR169] Zhao J, Sun Y, Shi P, Dong JN, Zuo LG, Wang HG, Gong JF, Li Y, Gu LL, Li N, Li JS, Zhu WM. Celastrol ameliorates experimental colitis in IL-10 deficient mice via the up-regulation of autophagy. Int Immunopharmacol. 2015;26:221–8. 25858875 10.1016/j.intimp.2015.03.033

[CR170] Zhao J, Dong JN, Wang HG, Zhao M, Sun J, Zhu WM, Zuo LG, Gong JF, Li Y, Gu LL, Li N, Li JS. Docosahexaenoic acid attenuated experimental chronic colitis in interleukin 10-deficient mice by enhancing autophagy through inhibition of the mTOR pathway. JPEN J Parenter Enteral Nutr. 2017;41:824–9. 26407598 10.1177/0148607115609308

[CR171] Zhao J, Wang H, Yang H, Zhou Y, Tang L. Autophagy induction by rapamycin ameliorates experimental colitis and improves intestinal epithelial barrier function in IL-10 knockout mice. Int Immunopharmacol. 2020a;81:105977. 31677991 10.1016/j.intimp.2019.105977

[CR172] Zhao ZQ, Liu WL, Guo SB, Bai R, Yan JL. Mechanism of methylprednisolone-induced primary cilia formation disorder and autophagy in osteoblasts. Orthop Surg. 2020b;12:645–52. 32064763 10.1111/os.12630 PMC7189053

[CR173] Zhou C, Ma K, Gao R, Mu C, Chen L, Liu Q, Luo Q, Feng D, Zhu Y, Chen Q. Regulation of mATG9 trafficking by Src- and ULK1-mediated phosphorylation in basal and starvation-induced autophagy. Cell Res. 2017;27:184–201. 27934868 10.1038/cr.2016.146 PMC5339848

